# A checklist for identifying determinants of practice: A systematic review and synthesis of frameworks and taxonomies of factors that prevent or enable improvements in healthcare professional practice

**DOI:** 10.1186/1748-5908-8-35

**Published:** 2013-03-23

**Authors:** Signe A Flottorp, Andrew D Oxman, Jane Krause, Nyokabi R Musila, Michel Wensing, Maciek Godycki-Cwirko, Richard Baker, Martin P Eccles

**Affiliations:** 1 Norwegian Knowledge Centre for the Health Services, Oslo, Norway; 2 Department of Public Health and Primary Health Care, University of Bergen, Bergen, Norway; 3 Department of Health Sciences, University of Leicester, Leicester, UK; 4 African Institute for Development Policy (AFIDEP), Nairobi, Kenya; 5 Scientific Institute for Quality of Healthcare, Nijmegen, Radboud University Nijmegen Medical Centre, Geert Grooteplein, Nijmegen, the Netherlands; 6 Department of Family and Community Medicine, Medical University of Lodz, Lodz, Poland; 7 Institute of Health and Society, Newcastle University, Baddiley-Clark Building, Richardson Road, Newcastle upon Tyne, UK

## Abstract

**Background:**

Determinants of practice are factors that might prevent or enable improvements. Several checklists, frameworks, taxonomies, and classifications of determinants of healthcare professional practice have been published. In this paper, we describe the development of a comprehensive, integrated checklist of determinants of practice (the TICD checklist).

**Methods:**

We performed a systematic review of frameworks of determinants of practice followed by a consensus process. We searched electronic databases and screened the reference lists of key background documents. Two authors independently assessed titles and abstracts, and potentially relevant full text articles. We compiled a list of attributes that a checklist should have: comprehensiveness, relevance, applicability, simplicity, logic, clarity, usability, suitability, and usefulness. We assessed included articles using these criteria and collected information about the theory, model, or logic underlying how the factors (determinants) were selected, described, and grouped, the strengths and weaknesses of the checklist, and the determinants and the domains in each checklist. We drafted a preliminary checklist based on an aggregated list of determinants from the included checklists, and finalized the checklist by a consensus process among implementation researchers.

**Results:**

We screened 5,778 titles and abstracts and retrieved 87 potentially relevant papers in full text. Several of these papers had references to papers that we also retrieved in full text. We also checked potentially relevant papers we had on file that were not retrieved by the searches. We included 12 checklists. None of these were completely comprehensive when compared to the aggregated list of determinants and domains. We developed a checklist with 57 potential determinants of practice grouped in seven domains: guideline factors, individual health professional factors, patient factors, professional interactions, incentives and resources, capacity for organisational change, and social, political, and legal factors. We also developed five worksheets to facilitate the use of the checklist.

**Conclusions:**

Based on a systematic review and a consensus process we developed a checklist that aims to be comprehensive and to build on the strengths of each of the 12 included checklists. The checklist is accompanied with five worksheets to facilitate its use in implementation research and quality improvement projects.

## Background

Tailored implementation interventions are strategies that are designed to achieve improvements in healthcare based on an assessment of determinants of practice. There is systematic review evidence that tailored interventions can improve healthcare [1], although the review identified that there was a clear need for an improved understanding of the methods of tailoring. Systematic tailoring entails (at least) three key steps: identification of the determinants of practice, designing implementation interventions appropriate to the determinants, and application and assessment of implementation interventions that are matched to the identified determinants [2].

The basic idea underlying tailored interventions is that different types and constellations of influences or determinants affect different types of practice changes. To implement an evidence-based recommendation in a specific context, we may need detailed information about how specific determinants might affect the desired changes in practice. The changes needed to implement different recommendations vary widely; some changes are relatively simple, while other changes are more complex; *e.g.*, requiring new ways of delivering or organising care. Different determinants are likely to affect different types of changes. The impact of a specific determinant for a specific type of change may vary across different contexts and different health professionals within a given context.

Determinants of healthcare professional practice are factors that might prevent or enable improvements in that practice. Such factors have also been referred to as barriers and enablers, barriers and facilitators, problems and needs, or disincentives and incentives [2]. Determinants of practice may act as moderators or ‘effect modifiers,’ or they may act as mediators; indicating that they are links in a chain of causal mechanism [3]. We have limited evidence on the moderating or mediating influence of specific determinants on the effectiveness of implementation interventions. It is unlikely that there is a simple linear causal relationship linking specific determinants to specific changes in practice; rather, it is more plausible that different determinants interact in ways that make it difficult to confidently predict the likely impact of each specific determinant.

Several checklists, frameworks, taxonomies, and classifications of determinants of practice have been published [3-8]. Some of these have been developed based on theories, and some based on empirical research.

Stavri and Michie have identified six different types of classification systems from the natural, medical, and social sciences: nomenclatures, hierarchical classification, matrix classification, ordered sets, faceted classification, and social categorization systems [9]. Different terms such as checklist, framework, taxonomy, and classifications may represent different ways of framing, understanding, or thinking about the idea of determinants of practice. However, for applied purposes they can also be considered to be similar tools. We use the term ‘checklist’ as a generic term for any system for identifying and classifying determinants of practice.

Having many such lists may be confusing both for those who use them as checklists for identifying determinants of practice and for those interpreting the results of studies that have used different checklists. A common checklist that can be used internationally across different settings and types of targeted practices should reduce this confusion and facilitate clear and consistent reporting and interpretation of implementation research. This would facilitate the design of effective interventions and accumulation of knowledge on change of healthcare practice.

We have developed a checklist to identify determinants of practice to be used and evaluated in the project ‘Tailored Implementation for Chronic Diseases’ (TICD) [2]. While our focus is thus primarily on healthcare for patients with chronic diseases, we suggest that the checklist can be applied more broadly.

The comprehensive, integrated checklist of determinants of practice (the TICD Checklist) is intended as a screening tool to identify determinants that warrant further in-depth investigation. Subsequent investigation of determinants and the design of implementation strategies should focus on the factors that are most relevant for a specific recommendation. The aim of the checklist is to guide reflection and data collection on determinants of practice for a particular change, in order to explore which specific influences are most likely to be important. The idea is that this can facilitate tailoring more effective change interventions and the evaluation and reporting of tailored interventions. The checklist is intended to help both implementation researchers and people responsible for quality improvement in healthcare.

In this paper we describe the development of the TICD checklist of determinants of healthcare professional practice and present the checklist and accompanying worksheets that we have developed to facilitate its use.

## Methods

Our objective was to identify or develop a list of determinants of practice that was comprehensive and non-overlapping. We also wanted it to be easy to understand and use.

### Study design

We performed a systematic review and synthesis of frameworks and taxonomies of determinants of practice, followed by a consensus process among implementation researchers to develop the TICD checklist.

### Review protocol and registration

This work is based on the protocol for the TICD project [2]. We developed, but did not publish or register a protocol for this systematic review of checklists of determinants of practice.

### Eligibility criteria

We included any kind of study or paper that described a checklist of determinants for changing healthcare professional practice, organisational change, or changes in health system arrangements. To be included, the checklist must have been used or been suitable for use in identifying determinants of practice prior to intervening to make improvements. We did not apply language restrictions.

We applied the following conceptual considerations when deciding on inclusion of studies in the review. Our focus was on determinants of change, including determinants of current practice that are relevant to achieving change. More specifically, we focussed on the implementation of evidence-based recommendations in health care. However, we also included checklists for the diffusion of innovations, if they met our other inclusion criteria described here. We defined ‘determinants of practice’ as factors that might prevent or enable healthcare improvements. These include factors that can be modified and factors that can be used to gauge the potential for achieving change. We considered evidence-based recommendations and innovations in any healthcare setting (including primary and secondary care) and in public health services as well as clinical services. Relevant outcomes included any desired change in the effectiveness, safety, efficiency, responsiveness, or equity of health services.

The determinants may be pragmatically defined or be linked to broader theoretical perspectives. They can relate to any or all of professional behaviour, organisation of healthcare, and health system arrangements. They can also be related to patient behaviours that might prevent or enable healthcare improvements and characteristics of the social and political environment, which might constrain or enable efforts to improve health services.

We excluded:

1. Checklists for determinants of health promotion (changing patient or health behaviours) and checklists that did not focus on health care.

2. Studies to identify barriers and enablers to inform the development of an intervention, (and not to develop a checklist to be used to identify barriers and enablers).

3. Checklists that were specific (and only applicable) for a particular type of practice or change.

4. Checklists that were narrowly focussed (*e.g.*, only focussed on a single domain, such as attributes of a guideline).

5. Frameworks that only included broad domains (*e.g.*, guideline factors) and not specific determinants within those domains (*e.g.*, clarity or cultural appropriateness).

### Search

We conducted two electronic searches. First, we used text words and index terms from published papers that we already had on file [3-8] to construct search strategies for Medline, the Cochrane Methodology Register, CINAHL, PsycInfo, Science Citation Index & Social Sciences Citation Index, and Sociological Abstracts. Second, in a parallel systematic review for the TICD project, we also searched for studies on methods for identifying barriers and enablers for changing practice, and we screened those search results for studies of checklists of determinants of practice. In the searches, we used terms such as barrier, obstacle, enabler, facilitator, classification, taxonomy, ontology, theory, and framework. The search strategies are reported in Additional file 1.

We screened the reference lists of key background documents and relevant articles, including a Cochrane review of tailored interventions [1].

### Study selection

Two authors (among SF, JK, NM and RB) independently read the titles and the abstracts resulting from the search process and eliminated any irrelevant articles. We retrieved the full text of potentially relevant articles. Two authors (among SF, AO, and JK) assessed each retrieved article using the selection criteria. We included articles meeting all of the selection criteria. Disagreements were resolved by discussion to reach consensus among the authors.

### Data collection process and data items

One author (SF, AO, or JK) collected the following information from each included article:

1. The theory, model or logic underlying how the determinants were selected, described and grouped (and relevant references).

2. Any information that was provided regarding the strengths or advantages and weaknesses or disadvantages of the checklist.

3. The determinants included in each checklist.

4. The domains (ways in which determinants were grouped) in each checklist.

A second author (SF, AO or JK) verified the data against the original articles.

### Synthesis of results

We compiled a list of attributes that a checklist of determinants of practice should have. We developed this list iteratively, building on previous criteria for ‘sensibility’ (the extent to which the criteria are sensible) [10-13], discussion amongst TICD collaborators, and iterative revisions. The list of attributes to be considered included:

1. Comprehensiveness: It should include all important domains and determinants.

2. Relevance: Irrelevant and unimportant domains and determinants should not be included.

3. Applicability: It should be applicable across different (relevant) settings, clinical and public health practice, and different types of targeted practices.

4. Simplicity: It should be as simple as possible.

5. Logic: The structure (the way in which determinants are organised) should be logical, easy to understand and easy to remember (for the intended target audience—people doing or using implementation research—including researchers, health professionals, and managers).

6. Clarity: Each dimension and determinant should be clearly labelled, defined and easy to understand.

7. Usability: It should be possible for researchers, health professionals and managers to use the checklist.

8. Suitability: It should be suitable both as a checklist for identifying determinants of practice and for reporting determinants of practice in studies and in systematic reviews.

9. Usefulness: It should be useful to people designing implementation strategies and reporting determinants of practice in research reports.

Three authors (SF, JK and AO) independently assessed the included checklists using the above criteria. Two authors (SF, AO) are physicians and one author (JK) is a dietician; all are researchers with strong commitment to evidence-based medicine and interest in how to implement this in practice. We also made an overall assessment of the checklists. This was a global judgement made independently by the three of us based on our assessments in relationship to each of the criteria that we used. Finally, we checked if there was anything else noteworthy that could be learned from the checklists, including how they were developed, evaluated, used, or presented. We identified additional desirable attributes of included checklists and then assessed whether each of the included checklists had each of the identified attributes.

### Development of a comprehensive checklist

One of the authors (AO) drafted a preliminary checklist based on the compiled list of determinants and domains from the included checklists, and this compiled list was checked by two authors (SF, JK). Determinants and domains that were identical or nearly identical were aggregated.

All of the authors gave feedback on the preliminary checklist. A revised draft was sent to all participants in the TICD project with a structured feedback form containing the desired attributes (Additional file 2). AO summarized the feedback, and revised the checklist accordingly. AO also drafted five worksheets to support use of the TICD checklist. The authors discussed the development of the checklist and worksheets in an informal and iterative process using telephone conferences and e-mails to reach a consensus on the determinants that should be included and the underlying model or logic that should be used to organise the determinants.

## Results

### Study selection

**Figure 1:**
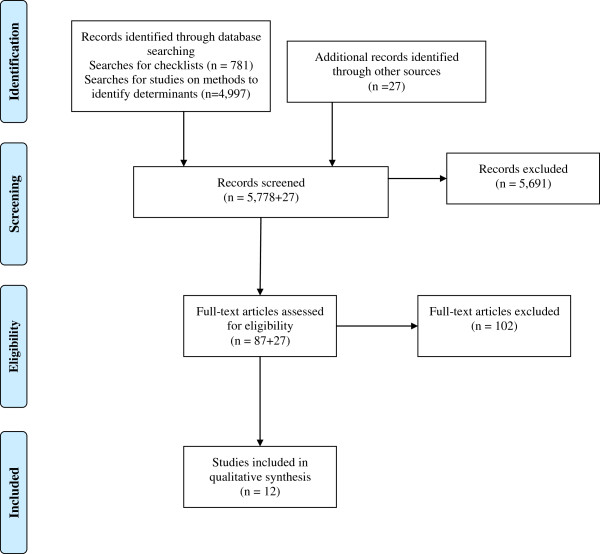
Study selection.

Our electronic searches for taxonomies or checklists for determinants of practice yielded 781 records. In addition, we had 4,997 records from searches for studies of methods for identifying barriers and enablers to changing practice. We screened all 5,778 titles and abstracts and retrieved 87 potentially relevant papers in full text. Several of these papers had references to papers that we also retrieved in full text and checked for inclusion. We also checked potentially relevant papers we had on file that were not retrieved by the searches. After excluding frameworks that were not comprehensive (*i.e.*, focussed on a specific domain or condition), we included 12 checklists of determinants of practice [3-5,7,8,14-20]. Details of the selection process are summarized in Figure 1.

### Study characteristics

The 12 included checklists varied in complexity and number of determinants, and in the way the determinants were grouped. Several of the included checklists were based on literature reviews [3,4,14,16,17,20]; others were mainly developed by brainstorming or consensus processes [5,7,15], and some of the papers did not specify the methods used to produce the checklists [8,19]. The PAHRIS framework was developed using retrospective case study analysis and concept analysis of each of the core elements evidence, context, and facilitation [21].

All of the checklists included determinants related to the individual professionals, such as knowledge, skills, and attitudes. Several of the checklists focussed on the individual health professionals. Six of the checklists included characteristics of the intervention, innovation, or guideline to be implemented. Determinants related to patients or recipients of care were included in five checklists. Professional interaction was explicitly addressed in four checklists. Determinants related to incentives and resources, capacity for organisational change, and social, political, and legal factors were listed in several of the checklists, but many determinants in these domains were lacking from several of the checklists, and they were not fully covered in any of them.

The domains and determinants extracted from the 12 checklists are presented in Additional file 3. Although we excluded checklists that we considered not comprehensive and not generic, which focussed on specific dimensions or conditions, none of the 12 included checklists were completely comprehensive when compared to the aggregated list of determinants and domains.

In addition to the determinants that we had compiled to assess the included checklists, we identified the following desirable attributes by examining the checklists:

1. Comprehensive with non-overlapping categories.

2. Easily understood and remembered.

3. Consistent framing of determinants and inclusion of both barriers and facilitators.

4. Inclusion of ‘Other (specify)’ in each domain (or where relevant) to capture determinants that might otherwise be missed [5].

5. Inclusion of:

a. Definitions or explanations [15,19].

b. Questions to be used in brainstorming/interview or surveys to identify determinants [7].

c. Methods to investigate each determinant (and why, based on empirical evidence and logic) [14].

d. Examples of specific determinants for specific recommendations [19].

e. Examples of implementation strategies to address each determinant [3,19,22].

6. Having a concise version (*e.g.*, domains and sub-domains) and a long (comprehensive) version.

7. Tools (worksheets and guides) for using the checklist [16,23].

8. A check regarding potential for perceptions to be wrong [4].

The first two of these attributes are similar to the criteria we used to assess the sensibility of the checklists (comprehensiveness and clarity). The others are desirable features that one or more of the included checklists had. They relate to the usability and usefulness of the checklists. None of the included checklists had all of these features.

There was little disagreement among the three authors assessing the included papers, and all disagreement was easily resolved by discussion.

### Synthesis of results—development of the TICD checklist of determinants of practice

Our checklist, based on the synthesis of the compiled list of determinants and domains, includes 57 potential determinants of practice grouped in seven domains. These seven domains are: guideline factors; individual health professional factors; patient factors; professional interactions; incentives and resources; capacity for organisational change; and social, political, and legal factors.

Table 1 summarizes the main domains and determinants in the included 12 checklists by comparison to the domains in the TICD checklist.

**Table 1:** The main domains in the 12 included checklists compared with the TICD checklist

**Included checklists (first author, year of publication, reference)**	**The seven domains of factors in the TICD checklist**						
	**Guideline factors**	**Individual health professional factors**	**Patient factors**	**Professional interactions**	**Incentives and resources**	**Capacity for organisational change**	**Social, political and legal factors**
Cabana 1999 [4]	**Behaviour - guideline factors** e.g. guideline characteristics and presence of conflicting guidelines	**Knowledge** e.g. familiarity, awareness **Attitudes** e.g. agreement with the specific guideline and guidelines in general, outcome expectancy, self-efficacy, motivation **Behaviour** e.g. external barriers, guideline factors, environmental factors	**Behaviour - external barriers** e.g. patient factors	Not included	**Behaviour - environmental factors,** lack of resources or reimbursement	**Behaviour - environmental factors** organisational constraints	Not included
Cochrane 2007 [14]	**Clinical practice guidelines/evidence barriers** e.g. utility, evidence, access, structure, applicability	**Cognitive/ behavioural barriers** e.g. knowledge, awareness, skill/ expertise **Health care professional barriers**	**Patient barriers** e.g. patient adherence	**Health care professional barriers** – e.g. peer influence	**Support/resources barriers** e.g. time, cost and funding issues, resources	**System/process barriers** e.g. organisational, system, workload/ overload	**Health care professional barriers** e.g. legal issues
Damschroder 2009 [15]	**Intervention characteristics** e.g. source, evidence strength and quality, relative advantage, adaptability, triability, complexity, cost	**Characteristics of individuals** e.g. knowledge and beliefs about the intervention, self-efficacy, individual stage of change, individual identification with organisation	**“Outer setting”** e.g. patient needs	**“Inner setting”** e.g. network and communications, culture	**“Inner setting”** e.g. available resources, incentives, access to knowledge and information **“Outer setting”** e.g. incentives	**“Inner setting”** e.g. learning climate, readiness for implementations, organisational incentives. **“Outer setting”** e.g. external policies **“Process”**	Not included
Greenhalgh 2004 [17]	**Innovation -** inherent attributes (e.g. relative advantage, compatibility, low complexity, trialability, observability), operational attributes (task relevance and usefulness, feasibility, nature of knowledge needed)	**Adopters and adoption -** characteristics and needs, meaning of the innovation, nature of the adoption decision, concerns	Not included	**Communication and influence** - nature of networks, main agents of social influence	**Inner context** organisation’s readiness (dedicated time/resources, broad based support)	**Inner context -** structural features of the organisation, organisation’s absorptive capacity for new knowledge, receptive context, organisation’s readiness **Outer context Implementation and sustainability External agencies**	Not included
Gurses 2010 [16]	**Guideline characteristics** e.g. relative advantage, compatibility, complexity, trialability, observability, strength of evidence	**Clinician characteristics** e.g. awareness, familiarity, agreement, self-efficacy, outcome expectancy, motivation, normative beliefs, subjective norms	Not included	**System characteristics** e.g. organisational characteristics (e.g. culture, teamwork, communication)	**Implementation characteristics** e.g. funding availability, monitoring and feedback mechanisms	**Implementation characteristics** e.g. tension for change, mandate/preparation-planning, leader and middle manager involvement and support, getting ideas from outside the organisation	Not included
Kitson 2008 [18]	**Evidence –** research and clinical experience	Not directly included	**Evidence –patient** experience	**Context** - context, culture,	**Context –** human/financial /technological/ equipment – resources appropriately allocated	**Context** - context, leadership, evaluation **Facilitation** - purpose, role, skills and attributes	Not included
Mäkelä 1999 [5]	Not included	**Professionals: Knowledge** – **Skills** – **Attitudes**	**Environment Social factors** support for or discourage-ment of change by others (e.g. patients)	**Environment Social factors** - support for or discouragement of change by others (e.g. colleagues at practice site, other members of professional team)	**Economic factors -** availability/lack of resources	**Organisational factors** – e.g. availability of guidelines at workplace, practicality within existing practice setting or routines, local infrastructures or rules)	Not included
Michie 2005 [7]	**Nature of the behaviours**	**Knowledge Skills Social/professional role and identity (self-standards) Beliefs about capabilities (self-efficacy) Beliefs about consequences (anticipated outcomes/attitude) Motivation and goals (intention) Memory, attention and decision processes Emotion Behavioural regulation**	Not included	**Social influences (norms)**	**Environmental context and resources (environmental constraints)**	**Environmental context and resources (environmental constraints)**	Not included
Rainbird 2006 [8]	**The innovation itself** - feasibility, credibility, accessibility, attractiveness	**Individual professional** - awareness, knowledge, attitude, motivation to change, behavioural routines	**Patient** - knowledge skills, attitude, compliance	**Social context** - opinion of colleagues, culture of the network, collaboration	**Organisational context** – capacities, resources	**Social context –** leadership **Organisational context** – care processes, staff, structures	**Economic and political context** - financial arrangements, regulations, policies
Saillour-Glenisson 2003 [20]	**Clinical practice guideline characteristics** - form, topic, compatibility, trialability, scientific basis, observability, adaptability, legal implications	**Physician characteristics -** knowledge about the CPG, attitude and agreement to CPG, psychological and socio-demographic and economic characteristics, job satisfaction, training	**Human environment** e.g. patient influence, attitude, physician - patient interaction	**Human environment** e.g. peer influence	**Oganizational environment –** financial context (resources)	**Organisational environment** (internal and external environment)	**Organisational environment –** External environment
SURE Collaboration 2011 [19]		**Providers of care** - knowledge and skills, attitudes regarding programme acceptability, appropriateness and credibility, motivation to change or adopt new behaviour	**Recipients of care –** knowledge, skills and attitudes, motivation to change or adopt new behaviour	**Other stakeholders** knowledge, skills, attitudes, motivation to change or adopt new behaviour	**Health system constraints** – e.g. accessibility of care, financial resources, human resources, incentives,	**Other stakeholders** Knowledge, skills, attitudes, motivation to change or adopt new behavior **Health system constraints** e.g. internal and external communication, management or leadership, information systems, patient flow processes, bureaucracy, relationship with norms and standards	**Social and political constraints -** ideology, short-term thinking, contracts, legislation or regulations, influential people, political stability
Wensing [3]	Not included	**Individual level: Cognitive factors Motivational factors Behavioural factors -**	Not included	**Professional interaction**: **Interaction in professional teams Structure of professional networks**	**Factors related to structures: Financial incentives** Regulations-purchaser provider contract relationships	**Organisational level: Organisational structures Organisational processes Organisational resources Factors related to structures: Societal factors**	**Factors related to structures: Regulations**

The full checklist includes a definition, exploratory questions and examples for each determinant (Additional file 4).

In the checklist, we have used the term ‘adherence’ (or ‘adhere’) as shorthand for adherence by health professionals to an evidence-based recommendation for clinical (or public health) practice. It should be noted, however, that we regard the use of evidence in clinical decisions and actions as more complex than simple ‘adherence.’ We have used ‘necessary change’ to refer to organisational or system changes that are needed to enable recommended clinical practice.

The focus of the checklist is on a specific recommendation for clinical practice. When multiple recommendations are being implemented, as in a clinical practice guideline, it is necessary to consider each determinant in relationship to each recommendation, because the relevance and importance of determinants can vary across different recommendations within a guideline.

### Worksheets

Based on ideas from the included checklists (particularly Barriers Identification and Mitigation Tool [23]), the drafts of the TICD checklist and feedback and discussion among the authors, we developed five worksheets that are intended to help people who are designing a tailored implementation strategy to apply the TICD checklist to identify determinants of practice for specific recommendations:

Worksheet 1 (Additional file 5) can be used to prioritize the recommendations that warrant targeted implementation efforts.

Worksheet 2 (Additional file 6) can be used to tailor the TICD checklist to address the specific recommendations that have been identified as priorities.

Worksheet 3 (Additional file 7) can be used to prioritize determinants based on the results of investigations (*e.g.*, brainstorming, surveys, interviews, or focus groups).

Worksheet 4 (Additional file 8) is to help people to develop an implementation strategy in a pragmatic way based flowing from the assessment of determinants.

Worksheet 5 (Additional file 9) is a suggested structure and table for reporting determinants in articles reporting trials or other evaluations of implementation strategies.

## Discussion

Using a systematic review and a consensus process in a group of implementation researchers, we have developed a comprehensive checklist for determinants of healthcare professional practice, and five worksheets to support the use of the checklist in implementation projects.

The checklist is comprehensive and generic. The checklist can potentially be used by both implementation researchers and others designing implementation interventions and quality improvement projects.

Unlike most systematic reviews, we did not assess the risk of bias in the methods used to develop included checklists, because this was not relevant to our review. However, we found that both methods based on theories and methods based on reviewing empirical studies resulted in incomplete lists of determinants. Instead, we applied criteria for assessing the ‘sensibility’ of the included checklists.

Also, unlike most systematic reviews, our synthesis did not focus on analysing the frequency with which different items were included (or any sort of an average across included checklists) or on the support for including (or excluding) different determinants provided for each of the included checklists. Instead, the synthesis of the included checklists was focussed on completeness (including all relevant determinants) without redundancy or undue overlap across included determinants, and grouping the determinants logically.

Given the complex, yet limited theoretical and empirical knowledge about determinants of practice in healthcare, based on several different scientific disciplines, it is difficult to develop a checklist that has all the attributes that we desired. Moreover, some of the desired attributes may need to be balanced against others. For example, the more comprehensive a checklist is, the less likely it is to be simple. None of the checklists we reviewed were comprehensive, compared to the compiled list of determinants based on all the 12 papers. The checklist we developed is more comprehensive, without containing overlapping determinants, but as a consequence it is less simple than some other checklists.

By developing a compiled list of determinants based on 12 different frameworks, we may have made a smorgasbord of a variety of issues and diluted the underlying conceptual or theoretical basis of the individual checklists we reviewed. However, all of the checklists included overlapping constructs and none had a compelling theoretical basis. Ultimately, the decision about how best to organise the checklist was based on our judgement that the checklist is organised in a way that is logical and easy to understand. This judgement was informed by our review and our experience as clinicians and researchers, and confirmed by feedback from others not involved in the development of the checklist.

Neither the checklist nor the worksheets have been validated. Further evaluation and possibly further modifications of the checklist and the worksheets are needed. This includes feedback from a broader range of people and application of the checklist in implementation studies, both of which are ongoing.

This work is based on a pragmatic approach to understanding health professional behaviour change. There are other paradigms, for example the social science interactionist paradigm that would not draw on the checklist approach we have devised.

The focus of the checklist is on provider behaviour, not patient behaviour. This might be considered a limitation of the review, as we are interested in implementing evidence-based practice. Our electronic searches did not identify all relevant checklists. Several of the included checklists were identified through screening of reference lists and by prior knowledge of the field. It is thus possible that we missed relevant papers. We are continuing to search for other checklists through contact with experts in the field and we hope that publicizing the checklist will encourage authors of relevant checklists to bring these to our attention. A list of excluded papers is available on request.

### Implications for research

The TICD checklist is not a finished product, and will benefit from further development. We have started using the checklist and worksheets in the TICD project, and we will systematically evaluate our experiences, with the aim of improving both the checklist and the worksheets. We have also contacted international experts about checklists that we might have missed and invited them to provide structured feedback on the checklist (Additional file 2).

We plan to update the checklist and worksheets based on a summary of the feedback from the international experts, assessments of potentially relevant papers that we might have missed, and an evaluation of our experiences having used the checklist in the TICD project.

We assume that assessments would be made within a time span where we would not anticipate differences due to changes over time. We would expect different assessors to have different perspectives and make different judgements. We are proposing a structured process that includes initial independent assessments, discussion about those assessments and setting priorities for collecting additional information to resolve disagreements or reduce uncertainty, and new assessments informed by the additional information that is collected (*e.g.*, from interviews or focus groups). We are using the checklist in this way and will report on this (and the advantages of different approaches to obtaining additional information) in subsequent papers.

We will assess if the checklist helps to identify important determinants of practice and to tailor effective implementation intervention. The checklist might also be used to test hypotheses in future research about whether some determinants are specific to some types of practice changes.

### Implications for practice

We have produced a comprehensive and generic checklist of determinants of practice, and five worksheets that can be used by people designing, carrying out, evaluating, and reporting implementation interventions. Although it can be used in practice, our testing of the checklist in practice is ongoing, and we anticipate further revisions of the checklist based on this experience.

With its seven domains and 57 determinants of practice, the checklist may be quite challenging to use even for experienced implementation researchers. For people involved in quality improvement, rather than research, it may be even more difficult to use such a long checklist, and a simpler version may be necessary.

## Conclusion

None of the checklists of determinants of healthcare professional practice that we found in our systematic review were comprehensive in comparison to a compiled list of determinants and dimensions. Furthermore, the 12 checklists included in our review each had advantages and disadvantages compared to each other. Based on our review of these checklists, we have developed a checklist that aims to be comprehensive and to build on the strengths of each of the checklists. We have developed five worksheets to facilitate the use of the resulting checklist in implementation research and quality improvement projects.

## Competing interests

ADO is a member of the Editorial Board and MPE and MW are Co-Editors in Chief of Implementation Science. Neither of the authors who are editors of Implementation Science were involved in the review of this paper, and all decisions on this manuscript were made by another senior editor.

## Authors’ contributions

Writing of the paper was led by SF with all authors commenting on drafts and approving the final version.

## Supplementary Material

Additional file 1 Search strategies. Click here for file

Additional file 2 Feedback on TICD Checklist. Click here for file

Additional file 3 Dimensions and factors from 12 comprehensive checklists. Click here for file

Additional file 4 TICD Checklist – definitions, questions and examples. Click here for file

Additional file 5 TICD Worksheet 1: Prioritisation of recommendations. Click here for file

Additional file 6 TICD Worksheet 2: Initial assessment of determinants. Click here for file

Additional file 7 TICD Worksheet 3: Prioritisation of determinants. Click here for file

Additional file 8 TICD Worksheet 4: Development of an implementation strategy. Click here for file

Additional file 9 TICD Worksheet 5: Reporting of determinants. Click here for file
