# Supplementary material for: A checklist for identifying determinants of practice: A systematic review and synthesis of frameworks and taxonomies of factors that prevent or enable improvements in healthcare professional practice
Source: Implement Sci. 2013 Mar 23;8:35. doi: 10.1186/1748-5908-8-35 (PMC3617095; doi:10.1186/1748-5908-8-35)
Supplement: Additional file 1 — Search strategies. [file 1748-5908-8-35-S1.pdf]

# Additional file 1 Search strategies

**We searched:**

- The Cochrane Central Register of Controlled Trials (CENTRAL) 2011, Issue 1, part of the *The Cochrane Library*. www.thecochranelibrary.com (DVD ROM), including the Cochrane Effective Practice and Organisation of Care (EPOC) Group Specialised Register, Wiley (searched 26 February 2011)
- Cochrane Methodology Register (CMR) 2011, Issue 1, part of the *The Cochrane Library*. www.thecochranelibrary.com (DVD ROM), Wiley (searched 26 February 2011)
- MEDLINE In-Process & Other Non-Indexed Citations and MEDLINE 1948 to present, Ovid (searched 25 February 2011)
- Cumulative Index to Nursing and Allied Health Literature 1981 to present (CINAHL), EbscoHost (searched 26 February 2011)
- PsycINFO 1967 to February Week 4 2011, Ovid (searched 25 February 2011)
- Sociological Abstracts 1952 to present, CSA Illumina (searched 26 March 2011)
- Science Citation Index & Social Sciences Citation Index 1975 to present, ISI Web of Science (searched 26 March 2011)

**Strategies**

**MEDLINE In-Process & Other Non-Indexed Citations and MEDLINE**

1. Communication Barriers/
2. ((barrier? or obstacle? or impediment? or hinder* or enabler? or facilitator? or moderator? or mediator? or driver? or modifier?) adj3 (identif* or elicit* or classif* or understand* or determin* or address* or examin* or study or pick up or look up or weed out or observ* or consider* or discover*)).tw.
3. or/1-2
4. Methods/
5. Observation/
6. Data Collection/
7. Health Surveys/
8. Interviews as Topic/
9. Narration/

10. Focus Groups/
11. Questionnaires/
12. Delphi Technique/
13. Records as Topic/
14. Dental Records/
15. Hospital Records/
16. Medical Records/
17. Nursing Records/
18. Qualitative Research/
19. Anthropology, Cultural/
20. Nursing Methodology Research/
21. Ethnology/
22. case reports.pt.
23. (method? or model? or technique? or strategy or strategies or interview* or focus group? or questionnaire? or questioning or data collection or client* record? or medical record? or dental record? or hospital record? or nursing record? or patient* record? or brainstorming or brain storming or informant? or discuss* or direct observation or participant observation or informal observation or survey? or nominal group? or qualitative research or grounded theory or qualitative study or qualitative studies or case study or case studies or case report? or anthropology or ethnology or ethnography or phenomenology or narrative? or narration?).tw.
24. or/4-23
25. Evidence-Based Practice/
26. Evidence-Based Dentistry/
27. Evidence-Based Medicine/
28. Evidence-Based Emergency Medicine/
29. Evidence-Based Nursing/
30. Advance Directive Adherence/
31. Guideline Adherence/
32. "Quality of Health Care"/
33. Quality Assurance, Health Care/
34. Quality Improvement/
35. "Diffusion of Innovation"/
36. Professional Competence/
37. Clinical Competence/

38. Physician's Practice Patterns/
39. Nurse's Practice Patterns/
40. Dentist's Practice Patterns/
41. (quality adj1 improv*).tw.
42. (quality adj3 (health care or healthcare or medical care or dental care or nursing care)).tw.
43. (improv* adj3 (health care or healthcare or medical care or dental care or nursing care or practice? or competence or prescri*)).tw.
44. (influence adj3 (health care or healthcare or medical care or dental care or nursing care or practice? or competence or prescri*)).tw.
45. ((clinical guideline? or practice guideline? or clinical path* or critical path*) adj3 (implement* or adher* or comply or compliance)).tw.
46. evidence based.tw.
47. ((tailor* or develop* or link*) adj3 intervention?).tw.
48. (knowledge adj (treasfer* or translat*)).tw.
49. or/25-48
50. 3 and 24 and 49
51. Classification/
52. (barrier? or obstacle? or impediment? or hinder* or enabler? or facilitator? or moderator? or mediator? or driver? or modifier?).tw.
53. 51 and 52
54. ((taxonomy or taxonomies or ontology or ontologies or framework? or frame work? or classify or classification? or theory or theories) adj3 (barrier? or obstacle? or impediment? or hinder* or enabler? or facilitator? or moderator? or mediator? or driver? or modifier?)).tw.
55. 53 or 54
56. 50 or 55

**PsycINFO**
1. Communication Barriers/
2. ((barrier? or obstacle? or impediment? or hinder* or enabler? or facilitator? or moderator? or mediator? or driver? or modifier?) adj3 (identif* or elicit* or classif* or understand* or determin* or address* or examin* or study or pick up or look up or weed out or observ* or consider* or discover*)).ti,ab.
3. or/1-2
4. Methodology/

5. Data Collection/
6. Interviews/
7. Questioning/
8. Group Discussion/
9. Questionnaires/
10. exp Surveys/
11. Qualitative Research/
12. Grounded Theory/
13. Observation Methods/
14. Case Report/
15. Client Records/
16. Medical Records/
17. Anthropology/
18. Ethnography/
19. Ethnology/
20. Phenomenology/
21. Narratives/
22. (field study or interview or focus group or literature review or nonclinical case study or qualitative study).md.
23. (method? or model? or technique? or strategy or strategies or interview* or focus group? or questionnaire? or questioning or data collection or client* record? or medical record? or dental record? or hospital record? or nursing record? or patient* record? or brainstorming or brain storming or informant? or discuss* or direct observation or participant observation or informal observation or survey? or nominal group? or qualitative research or grounded theory or qualitative study or qualitative studies or case study or case studies or case report? or anthropology or ethnology or ethnography or phenomenology or narrative? or narration?).ti,ab.
24. or/4-23
25. Evidence Based Practice/
26. Best Practices/
27. Professional Standards/
28. Professional Competence/
29. Employee Skills/
30. "Quality of Services"/

31. "Quality of Care"/
32. Knowledge Transfer/
33. (quality adj1 improv*).ti,ab.
34. (quality adj3 (health care or healthcare or medical care or dental care or nursing care)).ti,ab.
35. (improv* adj3 (health care or healthcare or medical care or dental care or nursing care or practice? or competence or prescri*)).ti,ab.
36. (influence adj3 (health care or healthcare or medical care or dental care or nursing care or practice? or competence or prescri*)).ti,ab.
37. ((clinical guideline? or practice guideline? or clinical path* or critical path*) adj3 (implement* or adher* or comply or compliance)).ti,ab.
38. evidence based.ti,ab.
39. ((tailor* or develop* or link*) adj3 intervention?).ti,ab.
40. (knowledge adj (treasfer* or translat*)).ti,ab.
41. or/25-40
42. 3 and 24 and 41
43. Taxonomies/
44. Ontologies/
45. 43 or 44
46. (barrier? or obstacle? or impediment? or hinder* or enabler? or facilitator? or moderator? or mediator? or driver? or modifier?).ti,ab.
47. 45 and 46
48. ((taxonomy or taxonomies or ontology or ontologies or framework? or frame work? or classify or classification? or theory or theories) adj3 (barrier? or obstacle? or impediment? or hinder* or enabler? or facilitator? or moderator? or mediator? or driver? or modifier?)).ti,ab.
49. 47 or 48
50. 42 or 49

**CINAHL (Methods)**

| | |
|---|---|
| S34 | S9 and S32 (Limiters - Exclude MEDLINE records) |
| S33 | S9 and S32 |
| S32 | S10 or S11 or S12 or S13 or S14 or S15 or S16 or S17 or S18 or S19 or S20 or S21 or S22 or S23 or S24 or S25 or S26 or S27 or S28 or S29 or S30 or S31 |

| | |
|---|---|
| S31 | TI ( (method* or model* or technique* or strategy or strategies or interview* or focus W0 group* or questionnaire* or questioning or "data collection" or client* W0 record* or medical W0 record* or dental W0 record* or hospital W0 record* or nursing W0 record* or patient* W0 record* or brainstorming or "brain storming" or informant* or discuss* or "direct observation" or "participant observation" or "informal observation" or survey* or nominal W0 group* or "qualitative research" or "grounded theory" or "qualitative study" or "qualitative studies" or "case study" or "case studies" or case W0 report* or anthropology or ethnology or ethnography or phenomenology or narrative* or narration*) ) or AB ( (method* or model* or technique* or strategy or strategies or interview* or focus W0 group* or questionnaire* or questioning or "data collection" or client* W0 record* or medical W0 record* or dental W0 record* or hospital W0 record* or nursing W0 record* or patient* W0 record* or brainstorming or "brain storming" or informant* or discuss* or "direct observation" or "participant observation" or "informal observation" or survey* or nominal W0 group* or "qualitative research" or "grounded theory" or "qualitative study" or "qualitative studies" or "case study" or "case studies" or case W0 report* or anthropology or ethnology or ethnography or phenomenology or narrative* or narration*) ) |
| S30 | (MH "Narratives") |
| S29 | (MH "Anthropology, Cultural") |
| S28 | (MH "Anthropology") |
| S27 | (MH Case Studies) |
| S26 | (MH Phenomenological Research) |
| S25 | (MH Naturalistic Inquiry) |
| S24 | (MH Grounded Theory) |
| S23 | (MH Ethnological Research) |
| S22 | (MH Ethnographic Research) |
| S21 | (MH Qualitative studies) |
| S20 | (MH Nursing Records) |
| S19 | (MH Medical Records) |
| S18 | (MH Surveys) |
| S17 | (MH Delphi Technique) |
| S16 | (MH "Questionnaires+") |
| S15 | (MH Focus Groups) |
| S14 | (MH Interviews) |
| S13 | (MH Data Collection Methods) |

| | |
|---|---|
| S12 | (MH Data Collection) |
| S11 | (MH Participant Observation) |
| S10 | (MH Observational Methods) |
| S9 | S1 or S2 or S3 or S4 or S5 or S6 or S7 or S8 |
| S8 | TI ( modifier* N3 identif* or modifier* N3 elicit* or modifier* N3 understand* ) or AB ( modifier* N3 identif* or modifier* N3 elicit* or modifier* N3 understand* ) |
| S7 | TI ( driver* N3 identif* or driver* N3 elicit* or driver* N3 understand* ) or AB ( driver* N3 identif* or driver* N3 elicit* or driver* N3 understand* ) |
| S6 | TI ( mediator* N3 identif* or mediator* N3 elicit* or mediator* N3 understand* ) or AB ( mediator* N3 identif* or mediator* N3 elicit* or mediator* N3 understand* ) |
| S5 | TI ( moderator* N3 identif* or moderator* N3 elicit* or moderator* N3 understand* ) or AB ( moderator* N3 identif* or moderator* N3 elicit* or moderator* N3 understand* ) |
| S4 | TI ( facilitator* N3 identif* or facilitator* N3 elicit* or facilitator* N3 understand* ) or AB ( facilitator* N3 identif* or facilitator* N3 elicit* or facilitator* N3 understand* ) |
| S3 | TI ( enabler* N3 identif* or enabler* N3 elicit* or enabler* N3 understand* ) or AB ( enabler* N3 identif* or enabler* N3 elicit* or enabler* N3 understand* ) |
| S2 | TI ( barrier* N3 identif* or barrier* N3 elicit* or barrier* N3 understand* ) or AB ( barrier* N3 identif* or barrier* N3 elicit* or barrier* N3 understand* ) |
| S1 | (MH "Communication Barriers") |

**CINAHL (Taxonomies)**

| | |
|---|---|
| S15 | S13 or S3<br>Limiters - Exclude MEDLINE records |
| S14 | S3 or S13 |
| S13 | S4 or S5 or S6 or S7 or S8 or S9 or S10 or S11 or S12 |
| S12 | TI ( theories N3 barrier* or theories N3 obstacle* or theories N3 impediment* or theories N3 hinder* or theories N3 enabler* or theories N3 facilitator* or theories N3 moderator* or theories N3 mediator* or theories N3 driver* or theories N3 modifier* ) or AU ( theories N3 barrier* or theories N3 obstacle* or theories N3 impediment* or theories N3 hinder* or theories N3 enabler* or theories N3 facilitator* or theories N3 moderator* or theories N3 mediator* or theories N3 driver* or theories N3 modifier* ) |
| S11 | TI ( theory N3 barrier* or theory N3 obstacle* or theory N3 impediment* or theory N3 hinder* or theory N3 enabler* or theory N3 facilitator* or theory N3 moderator* or theory N3 mediator* or theory N3 driver* or theory N3 modifier* ) or AB ( theory N3 barrier* or theory N3 obstacle* or theory N3 impediment* or theory N3 hinder* or |

| | |
|---|---|
| | theory N3 enabler* or theory N3 facilitator* or theory N3 moderator* or theory N3 mediator* or theory N3 driver* or theory N3 modifier* ) |
| S10 | TI ( classif* N3 barrier* or classif* N3 obstacle* or classif* N3 impediment* or classif* N3 hinder* or classif* N3 enabler* or classif* N3 facilitator* or classif* N3 moderator* or classif* N3 mediator* or classif* N3 driver* or classif* N3 modifier* ) or AB ( classif* N3 barrier* or classif* N3 obstacle* or classif* N3 impediment* or classif* N3 hinder* or classif* N3 enabler* or classif* N3 facilitator* or classif* N3 moderator* or classif* N3 mediator* or classif* N3 driver* or classif* N3 modifier* ) |
| S9 | TI ( "frame works" N3 barrier* or "frame works" N3 obstacle* or "frame works" N3 impediment* or "frame works" N3 hinder* or "frame works" N3 enabler* or "frame works" N3 facilitator* or "frame works" N3 moderator* or "frame works" N3 mediator* or "frame works" N3 driver* or "frame works" N3 modifier* ) or AB ( "frame works" N3 barrier* or "frame works" N3 obstacle* or "frame works" N3 impediment* or "frame works" N3 hinder* or "frame works" N3 enabler* or "frame works" N3 facilitator* or "frame works" N3 moderator* or "frame works" N3 mediator* or "frame works" N3 driver* or "frame works" N3 modifier* ) |
| S8 | TI ( "frame work" N3 barrier* or "frame work" N3 obstacle* or "frame work" N3 impediment* or "frame work" N3 hinder* or "frame work" N3 enabler* or "frame work" N3 facilitator* or "frame work" N3 moderator* or "frame work" N3 mediator* or "frame work" N3 driver* or "frame work" N3 modifier* ) or AB ( "frame work" N3 barrier* or "frame work" N3 obstacle* or "frame work" N3 impediment* or "frame work" N3 hinder* or "frame work" N3 enabler* or "frame work" N3 facilitator* or "frame work" N3 moderator* or "frame work" N3 mediator* or "frame work" N3 driver* or "frame work" N3 modifier* ) |
| S7 | TI ( framework* N3 barrier* or framework* N3 obstacle* or framework* N3 impediment* or framework* N3 hinder* or framework* N3 enabler* or framework* N3 facilitator* or framework* N3 moderator* or framework* N3 mediator* or framework* N3 driver* or framework* N3 modifier* ) or AB ( framework* N3 barrier* or framework* N3 obstacle* or framework* N3 impediment* or framework* N3 hinder* or framework* N3 enabler* or framework* N3 facilitator* or framework* N3 moderator* or framework* N3 mediator* or framework* N3 driver* or framework* N3 modifier* ) |
| S6 | TI ( ontologies N3 barrier* or ontologies N3 obstacle* or ontologies N3 impediment* or ontologies N3 hinder* or ontologies N3 enabler* or ontologies N3 facilitator* or ontologies N3 moderator* or ontologies N3 mediator* or ontologies N3 driver* or ontologies N3 modifier* ) or AB ( ontologies N3 barrier* or ontologies N3 obstacle* or ontologies N3 impediment* or ontologies N3 hinder* or ontologies N3 enabler* or ontologies N3 facilitator* or ontologies N3 moderator* or ontologies N3 mediator* or ontologies N3 driver* or ontologies N3 modifier* ) |
| S5 | TI ( ontology N3 barrier* or ontology N3 obstacle* or ontology N3 impediment* or ontology N3 hinder* or ontology N3 enabler* or ontology N3 facilitator* or ontology N3 moderator* or ontology N3 mediator* or ontology N3 driver* or ontology N3 modifier* ) or AB ( ontology N3 barrier* or ontology N3 obstacle* or ontology N3 impediment* or ontology N3 hinder* or ontology N3 enabler* or ontology N3 facilitator* or ontology N3 moderator* or ontology N3 mediator* or ontology N3 |

| | |
|---|---|
| | driver* or ontology N3 modifier* ) |
| S4 | TI ( taxonom* N3 barrier* or taxonom* N3 obstacle* or taxonom* N3 impediment* or taxonom* N3 hinder* or taxonom* N3 enabler* or taxonom* N3 facilitator* or taxonom* N3 moderator* or taxonom* N3 mediator* or taxonom* N3 driver* or taxonom* N3 modifier* ) or AB ( taxonom* N3 barrier* or taxonom* N3 obstacle* or taxonom* N3 impediment* or taxonom* N3 hinder* or taxonom* N3 enabler* or taxonom* N3 facilitator* or taxonom* N3 moderator* or taxonom* N3 mediator* or taxonom* N3 driver* or taxonom* N3 modifier* ) |
| S3 | S1 and S2 |
| S2 | TI ( barrier* or obstacle* or impediment* or hinder* or enabler* or facilitator* or moderator* or mediator* or driver* or modifier* ) or AB ( barrier* or obstacle* or impediment* or hinder* or enabler* or facilitator* or moderator* or mediator* or driver* or modifier* ) |
| S1 | (MH Classification) |

**CMR**

#1 ( (barrier* in Record Title near/3 identif* in Record Title) or (barrier* in Record Title near/3 elicit* in Record Title) or (barrier* in Record Title near/3 classif* in Record Title) or (barrier* in Record Title near/3 understand* in Record Title) or (barrier* in Record Title near/3 determin* in Record Title) or (barrier* in Record Title near/3 address* in Record Title) or (barrier* in Record Title near/3 examin* in Record Title) or (barrier* in Record Title near/3 study in Record Title) or (barrier* in Record Title near/3 observ* in Record Title) or (barrier* in Record Title near/3 consider* in Record Title) or (barrier* in Record Title near/3 discover* in Record Title) )

#2 ( (barrier* in Abstract near/3 identif* in Abstract) or (barrier* in Abstract near/3 elicit* in Abstract) or (barrier* in Abstract near/3 classif* in Abstract) or (barrier* in Abstract near/3 understand* in Abstract) or (barrier* in Abstract near/3 determin* in Abstract) or (barrier* in Abstract near/3 address* in Abstract) or (barrier* in Abstract near/3 examin* in Abstract) or (barrier* in Abstract near/3 study in Abstract) or (barrier* in Abstract near/3 observ* in Abstract) or (barrier* in Abstract near/3 consider* in Abstract) or (barrier* in Abstract near/3 discover* in Abstract) )

#3 ( (obstacle* in Record Title near/3 identif* in Record Title) or (obstacle* in Record Title near/3 elicit* in Record Title) or (obstacle* in Record Title near/3 classif* in Record Title) or (obstacle* in Record Title near/3 understand* in Record Title) or (obstacle* in Record Title near/3 determin* in Record Title) or (obstacle* in Record Title near/3 address* in Record Title) or (obstacle* in Record Title near/3 examin* in Record Title) or (obstacle* in Record Title near/3 study in Record Title) or (obstacle* in Record Title near/3 observ* in Record Title) or (obstacle* in Record Title near/3 consider* in Record Title) or (obstacle* in Record Title near/3 discover* in Record Title) )

#4 ( (obstacle* in Abstract near/3 identif* in Abstract) or (obstacle* in Abstract near/3

elicit* in Abstract) or (obstacle* in Abstract near/3 classif* in Abstract) or (obstacle* in Abstract near/3 understand* in Abstract) or (obstacle* in Abstract near/3 determin* in Abstract) or (obstacle* in Abstract near/3 address* in Abstract) or (obstacle* in Abstract near/3 examin* in Abstract) or (obstacle* in Abstract near/3 study in Abstract) or (obstacle* in Abstract near/3 observ* in Abstract) or (obstacle* in Abstract near/3 consider* in Abstract) or (obstacle* in Abstract near/3 discover* in Abstract) )

#5 ( (impediment* in Record Title near/3 identif* in Record Title) or (impediment* in Record Title near/3 elicit* in Record Title) or (impediment* in Record Title near/3 classif* in Record Title) or (impediment* in Record Title near/3 understand* in Record Title) or (impediment* in Record Title near/3 determin* in Record Title) or (impediment* in Record Title near/3 address* in Record Title) or (impediment* in Record Title near/3 examin* in Record Title) or (impediment* in Record Title near/3 study in Record Title) or (impediment* in Record Title near/3 observ* in Record Title) or (impediment* in Record Title near/3 consider* in Record Title) or (impediment* in Record Title near/3 discover* in Record Title) )

#6 ( (impediment* in Abstract near/3 identif* in Abstract) or (impediment* in Abstract near/3 elicit* in Abstract) or (impediment* in Abstract near/3 classif* in Abstract) or (impediment* in Abstract near/3 understand* in Abstract) or (impediment* in Abstract near/3 determin* in Abstract) or (impediment* in Abstract near/3 address* in Abstract) or (impediment* in Abstract near/3 examin* in Abstract) or (impediment* in Abstract near/3 study in Abstract) or (impediment* in Abstract near/3 observ* in Abstract) or (impediment* in Abstract near/3 consider* in Abstract) or (impediment* in Abstract near/3 discover* in Abstract) )

#7 ( (hinder* in Record Title near/3 identif* in Record Title) or (hinder* in Record Title near/3 elicit* in Record Title) or (hinder* in Record Title near/3 classif* in Record Title) or (hinder* in Record Title near/3 understand* in Record Title) or (hinder* in Record Title near/3 determin* in Record Title) or (hinder* in Record Title near/3 address* in Record Title) or (hinder* in Record Title near/3 examin* in Record Title) or (hinder* in Record Title near/3 study in Record Title) or (hinder* in Record Title near/3 observ* in Record Title) or (hinder* in Record Title near/3 consider* in Record Title) or (hinder* in Record Title near/3 discover* in Record Title) )

#8 ( (hinder* in Abstract near/3 identif* in Abstract) or (hinder* in Abstract near/3 elicit* in Abstract) or (hinder* in Abstract near/3 classif* in Abstract) or (hinder* in Abstract near/3 understand* in Abstract) or (hinder* in Abstract near/3 determin* in Abstract) or (hinder* in Abstract near/3 address* in Abstract) or (hinder* in Abstract near/3 examin* in Abstract) or (hinder* in Abstract near/3 study in Abstract) or (hinder* in Abstract near/3 observ* in Abstract) or (hinder* in Abstract near/3 consider* in Abstract) or (hinder* in Abstract near/3 discover* in Abstract) )

#9 ( (enabler* in Record Title near/3 identif* in Record Title) or (enabler* in Record Title near/3 elicit* in Record Title) or (enabler* in Record Title near/3 classif* in Record Title) or (enabler* in Record Title near/3 understand* in Record Title) or (enabler* in Record Title near/3 determin* in Record Title) or (enabler* in Record Title near/3 address* in Record Title) or (enabler* in Record Title near/3 examin* in Record Title) or (enabler* in Record Title near/3 study in Record Title) or (enabler* in Record Title near/3 observ* in Record Title) or (enabler* in Record Title near/3 consider* in Record Title) or (enabler* in Record Title near/3 discover*

in Record Title) )

#10 ( (enabler* in Abstract near/3 identif* in Abstract) or (enabler* in Abstract near/3 elicit* in Abstract) or (enabler* in Abstract near/3 classif* in Abstract) or (enabler* in Abstract near/3 understand* in Abstract) or (enabler* in Abstract near/3 determin* in Abstract) or (enabler* in Abstract near/3 address* in Abstract) or (enabler* in Abstract near/3 examin* in Abstract) or (enabler* in Abstract near/3 study in Abstract) or (enabler* in Abstract near/3 observ* in Abstract) or (enabler* in Abstract near/3 consider* in Abstract) or (enabler* in Abstract near/3 discover* in Abstract) )

#11 ( (facilitator* in Record Title near/3 identif* in Record Title) or (facilitator* in Record Title near/3 elicit* in Record Title) or (facilitator* in Record Title near/3 classif* in Record Title) or (facilitator* in Record Title near/3 understand* in Record Title) or (facilitator* in Record Title near/3 determin* in Record Title) or (facilitator* in Record Title near/3 address* in Record Title) or (facilitator* in Record Title near/3 examin* in Record Title) or (facilitator* in Record Title near/3 study in Record Title) or (facilitator* in Record Title near/3 observ* in Record Title) or (facilitator* in Record Title near/3 consider* in Record Title) or (facilitator* in Record Title near/3 discover* in Record Title) )

#12 ( (facilitator* in Abstract near/3 identif* in Abstract) or (facilitator* in Abstract near/3 elicit* in Abstract) or (facilitator* in Abstract near/3 classif* in Abstract) or (facilitator* in Abstract near/3 understand* in Abstract) or (facilitator* in Abstract near/3 determin* in Abstract) or (facilitator* in Abstract near/3 address* in Abstract) or (facilitator* in Abstract near/3 examin* in Abstract) or (facilitator* in Abstract near/3 study in Abstract) or (facilitator* in Abstract near/3 observ* in Abstract) or (facilitator* in Abstract near/3 consider* in Abstract) or (facilitator* in Abstract near/3 discover* in Abstract) )

#13 ( (moderator* in Record Title near/3 identif* in Record Title) or (moderator* in Record Title near/3 elicit* in Record Title) or (moderator* in Record Title near/3 classif* in Record Title) or (moderator* in Record Title near/3 understand* in Record Title) or (moderator* in Record Title near/3 determin* in Record Title) or (moderator* in Record Title near/3 address* in Record Title) or (moderator* in Record Title near/3 examin* in Record Title) or (moderator* in Record Title near/3 study in Record Title) or (moderator* in Record Title near/3 observ* in Record Title) or (moderator* in Record Title near/3 consider* in Record Title) or (moderator* in Record Title near/3 discover* in Record Title) )

#14 ( (moderator* in Abstract near/3 identif* in Abstract) or (moderator* in Abstract near/3 elicit* in Abstract) or (moderator* in Abstract near/3 classif* in Abstract) or (moderator* in Abstract near/3 understand* in Abstract) or (moderator* in Abstract near/3 determin* in Abstract) or (moderator* in Abstract near/3 address* in Abstract) or (moderator* in Abstract near/3 examin* in Abstract) or (moderator* in Abstract near/3 study in Abstract) or (moderator* in Abstract near/3 observ* in Abstract) or (moderator* in Abstract near/3 consider* in Abstract) or (moderator* in Abstract near/3 discover* in Abstract) )

#15 ( (mediator* in Record Title near/3 identif* in Record Title) or (mediator* in Record Title near/3 elicit* in Record Title) or (mediator* in Record Title near/3 classif* in Record Title) or (mediator* in Record Title near/3 understand* in Record Title) or (mediator* in Record Title near/3 determin* in Record Title) or

(mediator* in Record Title near/3 address* in Record Title) or (mediator* in Record Title near/3 examin* in Record Title) or (mediator* in Record Title near/3 study in Record Title) or (mediator* in Record Title near/3 observ* in Record Title) or (mediator* in Record Title near/3 consider* in Record Title) or (mediator* in Record Title near/3 discover* in Record Title) )

#16 ( (mediator* in Abstract near/3 identif* in Abstract) or (mediator* in Abstract near/3 elicit* in Abstract) or (mediator* in Abstract near/3 classif* in Abstract) or (mediator* in Abstract near/3 understand* in Abstract) or (mediator* in Abstract near/3 determin* in Abstract) or (mediator* in Abstract near/3 address* in Abstract) or (mediator* in Abstract near/3 examin* in Abstract) or (mediator* in Abstract near/3 study in Abstract) or (mediator* in Abstract near/3 observ* in Abstract) or (mediator* in Abstract near/3 consider* in Abstract) or (mediator* in Abstract near/3 discover* in Abstract) )

#17 ( (driver* in Record Title near/3 identif* in Record Title) or (driver* in Record Title near/3 elicit* in Record Title) or (driver* in Record Title near/3 classif* in Record Title) or (driver* in Record Title near/3 understand* in Record Title) or (driver* in Record Title near/3 determin* in Record Title) or (driver* in Record Title near/3 address* in Record Title) or (driver* in Record Title near/3 examin* in Record Title) or (driver* in Record Title near/3 study in Record Title) or (driver* in Record Title near/3 observ* in Record Title) or (driver* in Record Title near/3 consider* in Record Title) or (driver* in Record Title near/3 discover* in Record Title) )

#18 ( (driver* in Abstract near/3 identif* in Abstract) or (driver* in Abstract near/3 elicit* in Abstract) or (driver* in Abstract near/3 classif* in Abstract) or (driver* in Abstract near/3 understand* in Abstract) or (driver* in Abstract near/3 determin* in Abstract) or (driver* in Abstract near/3 address* in Abstract) or (driver* in Abstract near/3 examin* in Abstract) or (driver* in Abstract near/3 study in Abstract) or (driver* in Abstract near/3 observ* in Abstract) or (driver* in Abstract near/3 consider* in Abstract) or (driver* in Abstract near/3 discover* in Abstract) )

#19 ( (modifier* in Record Title near/3 identif* in Record Title) or (modifier* in Record Title near/3 elicit* in Record Title) or (modifier* in Record Title near/3 classif* in Record Title) or (modifier* in Record Title near/3 understand* in Record Title) or (modifier* in Record Title near/3 determin* in Record Title) or (modifier* in Record Title near/3 address* in Record Title) or (modifier* in Record Title near/3 examin* in Record Title) or (modifier* in Record Title near/3 study in Record Title) or (modifier* in Record Title near/3 observ* in Record Title) or (modifier* in Record Title near/3 consider* in Record Title) or (modifier* in Record Title near/3 discover* in Record Title) )

#20 ( (modifier* in Abstract near/3 identif* in Abstract) or (modifier* in Abstract near/3 elicit* in Abstract) or (modifier* in Abstract near/3 classif* in Abstract) or (modifier* in Abstract near/3 understand* in Abstract) or (modifier* in Abstract near/3 determin* in Abstract) or (modifier* in Abstract near/3 address* in Abstract) or (modifier* in Abstract near/3 examin* in Abstract) or (modifier* in Abstract near/3 study in Abstract) or (modifier* in Abstract near/3 observ* in Abstract) or (modifier* in Abstract near/3 consider* in Abstract) or (modifier* in Abstract near/3 discover* in Abstract) )

#21 (#1 or #2 or #3 or #4 or #5 or #6 or #7 or #8 or #9 or #10 or #11 or #12 or #13 or #14 or #15 or #16 or #17 or #18 or #19 or #20)

#22 ( (taxonom* in Record Title near/6 barrier* in Record Title) or (taxonom* in Record Title near/6 obstacle* in Record Title) or (taxonom* in Record Title near/6 impediment* in Record Title) or (taxonom* in Record Title near/6 hinder* in Record Title) or (taxonom* in Record Title near/6 enabler* in Record Title) or (taxonom* in Record Title near/6 facilitator* in Record Title) or (taxonom* in Record Title near/6 moderator* in Record Title) or (taxonom* in Record Title near/6 mediator* in Record Title) or (taxonom* in Record Title near/6 driver* in Record Title) or (taxonom* in Record Title near/6 modifier* in Record Title) )

#23 ( (taxonom* in Abstract near/6 barrier* in Abstract) or (taxonom* in Abstract near/6 obstacle* in Abstract) or (taxonom* in Abstract near/6 impediment* in Abstract) or (taxonom* in Abstract near/6 hinder* in Abstract) or (taxonom* in Abstract near/6 enabler* in Abstract) or (taxonom* in Abstract near/6 facilitator* in Abstract) or (taxonom* in Abstract near/6 moderator* in Abstract) or (taxonom* in Abstract near/6 mediator* in Abstract) or (taxonom* in Abstract near/6 driver* in Abstract) or (taxonom* in Abstract near/6 modifier* in Abstract) )

#24 ( (ontology in Record Title near/6 barrier* in Record Title) or (ontology in Record Title near/6 obstacle* in Record Title) or (ontology in Record Title near/6 impediment* in Record Title) or (ontology in Record Title near/6 hinder* in Record Title) or (ontology in Record Title near/6 enabler* in Record Title) or (ontology in Record Title near/6 facilitator* in Record Title) or (ontology in Record Title near/6 moderator* in Record Title) or (ontology in Record Title near/6 mediator* in Record Title) or (ontology in Record Title near/6 driver* in Record Title) or (ontology in Record Title near/6 modifier* in Record Title) )

#25 ( (ontology in Abstract near/6 barrier* in Abstract) or (ontology in Abstract near/6 obstacle* in Abstract) or (ontology in Abstract near/6 impediment* in Abstract) or (ontology in Abstract near/6 hinder* in Abstract) or (ontology in Abstract near/6 enabler* in Abstract) or (ontology in Abstract near/6 facilitator* in Abstract) or (ontology in Abstract near/6 moderator* in Abstract) or (ontology in Abstract near/6 mediator* in Abstract) or (ontology in Abstract near/6 driver* in Abstract) or (ontology in Abstract near/6 modifier* in Abstract) )

#26 ( (ontologies in Record Title near/6 barrier* in Record Title) or (ontologies in Record Title near/6 obstacle* in Record Title) or (ontologies in Record Title near/6 impediment* in Record Title) or (ontologies in Record Title near/6 hinder* in Record Title) or (ontologies in Record Title near/6 enabler* in Record Title) or (ontologies in Record Title near/6 facilitator* in Record Title) or (ontologies in Record Title near/6 moderator* in Record Title) or (ontologies in Record Title near/6 mediator* in Record Title) or (ontologies in Record Title near/6 driver* in Record Title) or (ontologies in Record Title near/6 modifier* in Record Title) )

#27 ( (ontologies in Abstract near/6 barrier* in Abstract) or (ontologies in Abstract near/6 obstacle* in Abstract) or (ontologies in Abstract near/6 impediment* in Abstract) or (ontologies in Abstract near/6 hinder* in Abstract) or (ontologies in Abstract near/6 enabler* in Abstract) or (ontologies in Abstract near/6 facilitator* in Abstract) or (ontologies in Abstract near/6 moderator* in Abstract) or (ontologies in Abstract near/6 mediator* in Abstract) or (ontologies in Abstract near/6 driver* in Abstract) or (ontologies in Abstract near/6 modifier* in Abstract) )

#28 ( (framework* in Record Title near/6 barrier* in Record Title) or (framework* in Record Title near/6 obstacle* in Record Title) or (framework* in Record Title

near/6 impediment* in Record Title) or (framework* in Record Title near/6 hinder* in Record Title) or (framework* in Record Title near/6 enabler* in Record Title) or (framework* in Record Title near/6 facilitator* in Record Title) or (framework* in Record Title near/6 moderator* in Record Title) or (framework* in Record Title near/6 mediator* in Record Title) or (framework* in Record Title near/6 driver* in Record Title) or (framework* in Record Title near/6 modifier* in Record Title) )

#29 ( (framework* in Abstract near/6 barrier* in Abstract) or (framework* in Abstract near/6 obstacle* in Abstract) or (framework* in Abstract near/6 impediment* in Abstract) or (framework* in Abstract near/6 hinder* in Abstract) or (framework* in Abstract near/6 enabler* in Abstract) or (framework* in Abstract near/6 facilitator* in Abstract) or (framework* in Abstract near/6 moderator* in Abstract) or (framework* in Abstract near/6 mediator* in Abstract) or (framework* in Abstract near/6 driver* in Abstract) or (framework* in Abstract near/6 modifier* in Abstract) )

#30 ( ("frame work*" in Record Title near/6 barrier* in Record Title) or ("frame work*" in Record Title near/6 obstacle* in Record Title) or ("frame work*" in Record Title near/6 impediment* in Record Title) or ("frame work*" in Record Title near/6 hinder* in Record Title) or ("frame work*" in Record Title near/6 enabler* in Record Title) or ("frame work*" in Record Title near/6 facilitator* in Record Title) or ("frame work*" in Record Title near/6 moderator* in Record Title) or ("frame work*" in Record Title near/6 mediator* in Record Title) or ("frame work*" in Record Title near/6 driver* in Record Title) or ("frame work*" in Record Title near/6 modifier* in Record Title) )

#31 ( ("frame work*" in Abstract near/6 barrier* in Abstract) or ("frame work*" in Abstract near/6 obstacle* in Abstract) or ("frame work*" in Abstract near/6 impediment* in Abstract) or ("frame work*" in Abstract near/6 hinder* in Abstract) or ("frame work*" in Abstract near/6 enabler* in Abstract) or ("frame work*" in Abstract near/6 facilitator* in Abstract) or ("frame work*" in Abstract near/6 moderator* in Abstract) or ("frame work*" in Abstract near/6 mediator* in Abstract) or ("frame work*" in Abstract near/6 driver* in Abstract) or ("frame work*" in Abstract near/6 modifier* in Abstract) )

#32 ( (classify in Record Title near/6 barrier* in Record Title) or (classify in Record Title near/6 obstacle* in Record Title) or (classify in Record Title near/6 impediment* in Record Title) or (classify in Record Title near/6 hinder* in Record Title) or (classify in Record Title near/6 enabler* in Record Title) or (classify in Record Title near/6 facilitator* in Record Title) or (classify in Record Title near/6 moderator* in Record Title) or (classify in Record Title near/6 mediator* in Record Title) or (classify in Record Title near/6 driver* in Record Title) or (classify in Record Title near/6 modifier* in Record Title) )

#33 ( (classify in Abstract near/6 barrier* in Abstract) or (classify in Abstract near/6 obstacle* in Abstract) or (classify in Abstract near/6 impediment* in Abstract) or (classify in Abstract near/6 hinder* in Abstract) or (classify in Abstract near/6 enabler* in Abstract) or (classify in Abstract near/6 facilitator* in Abstract) or (classify in Abstract near/6 moderator* in Abstract) or (classify in Abstract near/6 mediator* in Abstract) or (classify in Abstract near/6 driver* in Abstract) or (classify in Abstract near/6 modifier* in Abstract) )

#34 ( (classification* in Record Title near/6 barrier* in Record Title) or (classification*

in Record Title near/6 obstacle* in Record Title) or (classification* in Record Title near/6 impediment* in Record Title) or (classification* in Record Title near/6 hinder* in Record Title) or (classification* in Record Title near/6 enabler* in Record Title) or (classification* in Record Title near/6 facilitator* in Record Title) or (classification* in Record Title near/6 moderator* in Record Title) or (classification* in Record Title near/6 mediator* in Record Title) or (classification* in Record Title near/6 driver* in Record Title) or (classification* in Record Title near/6 modifier* in Record Title) )

#35 ( (classification* in Abstract near/6 barrier* in Abstract) or (classification* in Abstract near/6 obstacle* in Abstract) or (classification* in Abstract near/6 impediment* in Abstract) or (classification* in Abstract near/6 hinder* in Abstract) or (classification* in Abstract near/6 enabler* in Abstract) or (classification* in Abstract near/6 facilitator* in Abstract) or (classification* in Abstract near/6 moderator* in Abstract) or (classification* in Abstract near/6 mediator* in Abstract) or (classification* in Abstract near/6 driver* in Abstract) or (classification* in Abstract near/6 modifier* in Abstract) )

#36 ( (theory in Record Title near/6 barrier* in Record Title) or (theory in Record Title near/6 obstacle* in Record Title) or (theory in Record Title near/6 impediment* in Record Title) or (theory in Record Title near/6 hinder* in Record Title) or (theory in Record Title near/6 enabler* in Record Title) or (theory in Record Title near/6 facilitator* in Record Title) or (theory in Record Title near/6 moderator* in Record Title) or (theory in Record Title near/6 mediator* in Record Title) or (theory in Record Title near/6 driver* in Record Title) or (theory in Record Title near/6 modifier* in Record Title) )

#37 ( (theory in Abstract near/6 barrier* in Abstract) or (theory in Abstract near/6 obstacle* in Abstract) or (theory in Abstract near/6 impediment* in Abstract) or (theory in Abstract near/6 hinder* in Abstract) or (theory in Abstract near/6 enabler* in Abstract) or (theory in Abstract near/6 facilitator* in Abstract) or (theory in Abstract near/6 moderator* in Abstract) or (theory in Abstract near/6 mediator* in Abstract) or (theory in Abstract near/6 driver* in Abstract) or (theory in Abstract near/6 modifier* in Abstract) )

#38 ( (theories in Record Title near/6 barrier* in Record Title) or (theories in Record Title near/6 obstacle* in Record Title) or (theories in Record Title near/6 impediment* in Record Title) or (theories in Record Title near/6 hinder* in Record Title) or (theories in Record Title near/6 enabler* in Record Title) or (theories in Record Title near/6 facilitator* in Record Title) or (theories in Record Title near/6 moderator* in Record Title) or (theories in Record Title near/6 mediator* in Record Title) or (theories in Record Title near/6 driver* in Record Title) or (theories in Record Title near/6 modifier* in Record Title) )

#39 ( (theories in Abstract near/6 barrier* in Abstract) or (theories in Abstract near/6 obstacle* in Abstract) or (theories in Abstract near/6 impediment* in Abstract) or (theories in Abstract near/6 hinder* in Abstract) or (theories in Abstract near/6 enabler* in Abstract) or (theories in Abstract near/6 facilitator* in Abstract) or (theories in Abstract near/6 moderator* in Abstract) or (theories in Abstract near/6 mediator* in Abstract) or (theories in Abstract near/6 driver* in Abstract) or (theories in Abstract near/6 modifier* in Abstract) )

#40 (#22 or #23 or #24 or #25 or #26 or #27 or #28 or #29 or #30 or #31 or #32 or #33

or #34 or #35 or #36 or #37 or #38 or #39)

#41 (#21 or #40)

**EPOC Register**

#1 ( (barrier* in Record Title near/3 identif* in Record Title) or (barrier* in Record Title near/3 elicit* in Record Title) or (barrier* in Record Title near/3 classif* in Record Title) or (barrier* in Record Title near/3 understand* in Record Title) or (barrier* in Record Title near/3 determin* in Record Title) or (barrier* in Record Title near/3 address* in Record Title) or (barrier* in Record Title near/3 examin* in Record Title) or (barrier* in Record Title near/3 study in Record Title) or (barrier* in Record Title near/3 observ* in Record Title) or (barrier* in Record Title near/3 consider* in Record Title) or (barrier* in Record Title near/3 discover* in Record Title) )

#2 ( (barrier* in Abstract near/3 identif* in Abstract) or (barrier* in Abstract near/3 elicit* in Abstract) or (barrier* in Abstract near/3 classif* in Abstract) or (barrier* in Abstract near/3 understand* in Abstract) or (barrier* in Abstract near/3 determin* in Abstract) or (barrier* in Abstract near/3 address* in Abstract) or (barrier* in Abstract near/3 examin* in Abstract) or (barrier* in Abstract near/3 study in Abstract) or (barrier* in Abstract near/3 observ* in Abstract) or (barrier* in Abstract near/3 consider* in Abstract) or (barrier* in Abstract near/3 discover* in Abstract) )

#3 ( (obstacle* in Record Title near/3 identif* in Record Title) or (obstacle* in Record Title near/3 elicit* in Record Title) or (obstacle* in Record Title near/3 classif* in Record Title) or (obstacle* in Record Title near/3 understand* in Record Title) or (obstacle* in Record Title near/3 determin* in Record Title) or (obstacle* in Record Title near/3 address* in Record Title) or (obstacle* in Record Title near/3 examin* in Record Title) or (obstacle* in Record Title near/3 study in Record Title) or (obstacle* in Record Title near/3 observ* in Record Title) or (obstacle* in Record Title near/3 consider* in Record Title) or (obstacle* in Record Title near/3 discover* in Record Title) )

#4 ( (obstacle* in Abstract near/3 identif* in Abstract) or (obstacle* in Abstract near/3 elicit* in Abstract) or (obstacle* in Abstract near/3 classif* in Abstract) or (obstacle* in Abstract near/3 understand* in Abstract) or (obstacle* in Abstract near/3 determin* in Abstract) or (obstacle* in Abstract near/3 address* in Abstract) or (obstacle* in Abstract near/3 examin* in Abstract) or (obstacle* in Abstract near/3 study in Abstract) or (obstacle* in Abstract near/3 observ* in Abstract) or (obstacle* in Abstract near/3 consider* in Abstract) or (obstacle* in Abstract near/3 discover* in Abstract) )

#5 ( (impediment* in Record Title near/3 identif* in Record Title) or (impediment* in Record Title near/3 elicit* in Record Title) or (impediment* in Record Title near/3 classif* in Record Title) or (impediment* in Record Title near/3 understand* in Record Title) or (impediment* in Record Title near/3 determin* in Record Title) or (impediment* in Record Title near/3 address* in Record Title) or (impediment* in Record Title near/3 examin* in Record Title) or (impediment* in Record Title near/3 study in Record Title) or (impediment* in Record Title near/3 observ* in Record Title) or (impediment* in Record Title near/3 consider* in Record Title) or (impediment* in Record Title near/3 discover* in Record Title) )

#6 ( (impediment* in Abstract near/3 identif* in Abstract) or (impediment* in Abstract near/3 elicit* in Abstract) or (impediment* in Abstract near/3 classif* in Abstract) or (impediment* in Abstract near/3 understand* in Abstract) or (impediment* in Abstract near/3 determin* in Abstract) or (impediment* in Abstract near/3 address* in Abstract) or (impediment* in Abstract near/3 examin* in Abstract) or (impediment* in Abstract near/3 study in Abstract) or (impediment* in Abstract near/3 observ* in Abstract) or (impediment* in Abstract near/3 consider* in Abstract) or (impediment* in Abstract near/3 discover* in Abstract) )

#7 ( (hinder* in Record Title near/3 identif* in Record Title) or (hinder* in Record Title near/3 elicit* in Record Title) or (hinder* in Record Title near/3 classif* in Record Title) or (hinder* in Record Title near/3 understand* in Record Title) or (hinder* in Record Title near/3 determin* in Record Title) or (hinder* in Record Title near/3 address* in Record Title) or (hinder* in Record Title near/3 examin* in Record Title) or (hinder* in Record Title near/3 study in Record Title) or (hinder* in Record Title near/3 observ* in Record Title) or (hinder* in Record Title near/3 consider* in Record Title) or (hinder* in Record Title near/3 discover* in Record Title) )

#8 ( (hinder* in Abstract near/3 identif* in Abstract) or (hinder* in Abstract near/3 elicit* in Abstract) or (hinder* in Abstract near/3 classif* in Abstract) or (hinder* in Abstract near/3 understand* in Abstract) or (hinder* in Abstract near/3 determin* in Abstract) or (hinder* in Abstract near/3 address* in Abstract) or (hinder* in Abstract near/3 examin* in Abstract) or (hinder* in Abstract near/3 study in Abstract) or (hinder* in Abstract near/3 observ* in Abstract) or (hinder* in Abstract near/3 consider* in Abstract) or (hinder* in Abstract near/3 discover* in Abstract) )

#9 ( (enabler* in Record Title near/3 identif* in Record Title) or (enabler* in Record Title near/3 elicit* in Record Title) or (enabler* in Record Title near/3 classif* in Record Title) or (enabler* in Record Title near/3 understand* in Record Title) or (enabler* in Record Title near/3 determin* in Record Title) or (enabler* in Record Title near/3 address* in Record Title) or (enabler* in Record Title near/3 examin* in Record Title) or (enabler* in Record Title near/3 study in Record Title) or (enabler* in Record Title near/3 observ* in Record Title) or (enabler* in Record Title near/3 consider* in Record Title) or (enabler* in Record Title near/3 discover* in Record Title) )

#10 ( (enabler* in Abstract near/3 identif* in Abstract) or (enabler* in Abstract near/3 elicit* in Abstract) or (enabler* in Abstract near/3 classif* in Abstract) or (enabler* in Abstract near/3 understand* in Abstract) or (enabler* in Abstract near/3 determin* in Abstract) or (enabler* in Abstract near/3 address* in Abstract) or (enabler* in Abstract near/3 examin* in Abstract) or (enabler* in Abstract near/3 study in Abstract) or (enabler* in Abstract near/3 observ* in Abstract) or (enabler* in Abstract near/3 consider* in Abstract) or (enabler* in Abstract near/3 discover* in Abstract) )

#11 ( (facilitator* in Record Title near/3 identif* in Record Title) or (facilitator* in Record Title near/3 elicit* in Record Title) or (facilitator* in Record Title near/3 classif* in Record Title) or (facilitator* in Record Title near/3 understand* in Record Title) or (facilitator* in Record Title near/3 determin* in Record Title) or (facilitator* in Record Title near/3 address* in Record Title) or (facilitator* in Record Title near/3 examin* in Record Title) or (facilitator* in Record Title near/3 study in Record Title) or (facilitator* in Record Title near/3 observ* in Record Title) or (facilitator* in Record Title near/3 consider* in Record Title) or (facilitator* in Record Title near/3 discover* in Record Title) )

#12 ( (facilitator* in Abstract near/3 identif* in Abstract) or (facilitator* in Abstract near/3 elicit* in Abstract) or (facilitator* in Abstract near/3 classif* in Abstract) or (facilitator* in Abstract near/3 understand* in Abstract) or (facilitator* in Abstract near/3 determin* in Abstract) or (facilitator* in Abstract near/3 address* in Abstract) or (facilitator* in Abstract near/3 examin* in Abstract) or (facilitator* in Abstract near/3 study in Abstract) or (facilitator* in Abstract near/3 observ* in Abstract) or (facilitator* in Abstract near/3 consider* in Abstract) or (facilitator* in Abstract near/3 discover* in Abstract) )

#13 ( (moderator* in Record Title near/3 identif* in Record Title) or (moderator* in Record Title near/3 elicit* in Record Title) or (moderator* in Record Title near/3 classif* in Record Title) or (moderator* in Record Title near/3 understand* in Record Title) or (moderator* in Record Title near/3 determin* in Record Title) or (moderator* in Record Title near/3 address* in Record Title) or (moderator* in Record Title near/3 examin* in Record Title) or (moderator* in Record Title near/3 study in Record Title) or (moderator* in Record Title near/3 observ* in Record Title) or (moderator* in Record Title near/3 consider* in Record Title) or (moderator* in Record Title near/3 discover* in Record Title) )

#14 ( (moderator* in Abstract near/3 identif* in Abstract) or (moderator* in Abstract near/3 elicit* in Abstract) or (moderator* in Abstract near/3 classif* in Abstract) or (moderator* in Abstract near/3 understand* in Abstract) or (moderator* in Abstract near/3 determin* in Abstract) or (moderator* in Abstract near/3 address* in Abstract) or (moderator* in Abstract near/3 examin* in Abstract) or (moderator* in Abstract near/3 study in Abstract) or (moderator* in Abstract near/3 observ* in Abstract) or (moderator* in Abstract near/3 consider* in Abstract) or (moderator* in Abstract near/3 discover* in Abstract) )

#15 ( (mediator* in Record Title near/3 identif* in Record Title) or (mediator* in Record Title near/3 elicit* in Record Title) or (mediator* in Record Title near/3 classif* in Record Title) or (mediator* in Record Title near/3 understand* in Record Title) or (mediator* in Record Title near/3 determin* in Record Title) or (mediator* in Record Title near/3 address* in Record Title) or (mediator* in Record Title near/3 examin* in Record Title) or (mediator* in Record Title near/3 study in Record Title) or (mediator* in Record Title near/3 observ* in Record Title) or (mediator* in Record Title near/3 consider* in Record Title) or (mediator* in Record Title near/3 discover* in Record Title) )

#16 ( (mediator* in Abstract near/3 identif* in Abstract) or (mediator* in Abstract near/3 elicit* in Abstract) or (mediator* in Abstract near/3 classif* in Abstract) or (mediator* in Abstract near/3 understand* in Abstract) or (mediator* in Abstract near/3 determin* in Abstract) or (mediator* in Abstract near/3 address* in Abstract) or (mediator* in Abstract near/3 examin* in Abstract) or (mediator* in Abstract near/3 study in Abstract) or (mediator* in Abstract near/3 observ* in Abstract) or (mediator* in Abstract near/3 consider* in Abstract) or (mediator* in Abstract near/3 discover* in Abstract) )

#17 ( (driver* in Record Title near/3 identif* in Record Title) or (driver* in Record Title near/3 elicit* in Record Title) or (driver* in Record Title near/3 classif* in Record Title) or (driver* in Record Title near/3 understand* in Record Title) or (driver* in Record Title near/3 determin* in Record Title) or (driver* in Record Title near/3 address* in Record Title) or (driver* in Record Title near/3 examin* in Record Title) or (driver* in Record Title near/3 study in Record Title) or (driver* in Record Title

near/3 observ* in Record Title) or (driver* in Record Title near/3 consider* in Record Title) or (driver* in Record Title near/3 discover* in Record Title) )

#18 ( (driver* in Abstract near/3 identif* in Abstract) or (driver* in Abstract near/3 elicit* in Abstract) or (driver* in Abstract near/3 classif* in Abstract) or (driver* in Abstract near/3 understand* in Abstract) or (driver* in Abstract near/3 determin* in Abstract) or (driver* in Abstract near/3 address* in Abstract) or (driver* in Abstract near/3 examin* in Abstract) or (driver* in Abstract near/3 study in Abstract) or (driver* in Abstract near/3 observ* in Abstract) or (driver* in Abstract near/3 consider* in Abstract) or (driver* in Abstract near/3 discover* in Abstract) )

#19 ( (modifier* in Record Title near/3 identif* in Record Title) or (modifier* in Record Title near/3 elicit* in Record Title) or (modifier* in Record Title near/3 classif* in Record Title) or (modifier* in Record Title near/3 understand* in Record Title) or (modifier* in Record Title near/3 determin* in Record Title) or (modifier* in Record Title near/3 address* in Record Title) or (modifier* in Record Title near/3 examin* in Record Title) or (modifier* in Record Title near/3 study in Record Title) or (modifier* in Record Title near/3 observ* in Record Title) or (modifier* in Record Title near/3 consider* in Record Title) or (modifier* in Record Title near/3 discover* in Record Title) )

#20 ( (modifier* in Abstract near/3 identif* in Abstract) or (modifier* in Abstract near/3 elicit* in Abstract) or (modifier* in Abstract near/3 classif* in Abstract) or (modifier* in Abstract near/3 understand* in Abstract) or (modifier* in Abstract near/3 determin* in Abstract) or (modifier* in Abstract near/3 address* in Abstract) or (modifier* in Abstract near/3 examin* in Abstract) or (modifier* in Abstract near/3 study in Abstract) or (modifier* in Abstract near/3 observ* in Abstract) or (modifier* in Abstract near/3 consider* in Abstract) or (modifier* in Abstract near/3 discover* in Abstract) )

#21 (#1 or #2 or #3 or #4 or #5 or #6 or #7 or #8 or #9 or #10 or #11 or #12 or #13 or #14 or #15 or #16 or #17 or #18 or #19 or #20)

#22 ( (taxonom* in Record Title near/6 barrier* in Record Title) or (taxonom* in Record Title near/6 obstacle* in Record Title) or (taxonom* in Record Title near/6 impediment* in Record Title) or (taxonom* in Record Title near/6 hinder* in Record Title) or (taxonom* in Record Title near/6 enabler* in Record Title) or (taxonom* in Record Title near/6 facilitator* in Record Title) or (taxonom* in Record Title near/6 moderator* in Record Title) or (taxonom* in Record Title near/6 mediator* in Record Title) or (taxonom* in Record Title near/6 driver* in Record Title) or (taxonom* in Record Title near/6 modifier* in Record Title) )

#23 ( (taxonom* in Abstract near/6 barrier* in Abstract) or (taxonom* in Abstract near/6 obstacle* in Abstract) or (taxonom* in Abstract near/6 impediment* in Abstract) or (taxonom* in Abstract near/6 hinder* in Abstract) or (taxonom* in Abstract near/6 enabler* in Abstract) or (taxonom* in Abstract near/6 facilitator* in Abstract) or (taxonom* in Abstract near/6 moderator* in Abstract) or (taxonom* in Abstract near/6 mediator* in Abstract) or (taxonom* in Abstract near/6 driver* in Abstract) or (taxonom* in Abstract near/6 modifier* in Abstract) )

#24 ( (ontology in Record Title near/6 barrier* in Record Title) or (ontology in Record Title near/6 obstacle* in Record Title) or (ontology in Record Title near/6 impediment* in Record Title) or (ontology in Record Title near/6 hinder* in Record Title) or (ontology in Record Title near/6 enabler* in Record Title) or (ontology in

Record Title near/6 facilitator* in Record Title) or (ontology in Record Title near/6 moderator* in Record Title) or (ontology in Record Title near/6 mediator* in Record Title) or (ontology in Record Title near/6 driver* in Record Title) or (ontology in Record Title near/6 modifier* in Record Title) )

#25 ( (ontology in Abstract near/6 barrier* in Abstract) or (ontology in Abstract near/6 obstacle* in Abstract) or (ontology in Abstract near/6 impediment* in Abstract) or (ontology in Abstract near/6 hinder* in Abstract) or (ontology in Abstract near/6 enabler* in Abstract) or (ontology in Abstract near/6 facilitator* in Abstract) or (ontology in Abstract near/6 moderator* in Abstract) or (ontology in Abstract near/6 mediator* in Abstract) or (ontology in Abstract near/6 driver* in Abstract) or (ontology in Abstract near/6 modifier* in Abstract) )

#26 ( (ontologies in Record Title near/6 barrier* in Record Title) or (ontologies in Record Title near/6 obstacle* in Record Title) or (ontologies in Record Title near/6 impediment* in Record Title) or (ontologies in Record Title near/6 hinder* in Record Title) or (ontologies in Record Title near/6 enabler* in Record Title) or (ontologies in Record Title near/6 facilitator* in Record Title) or (ontologies in Record Title near/6 moderator* in Record Title) or (ontologies in Record Title near/6 mediator* in Record Title) or (ontologies in Record Title near/6 driver* in Record Title) or (ontologies in Record Title near/6 modifier* in Record Title) )

#27 ( (ontologies in Abstract near/6 barrier* in Abstract) or (ontologies in Abstract near/6 obstacle* in Abstract) or (ontologies in Abstract near/6 impediment* in Abstract) or (ontologies in Abstract near/6 hinder* in Abstract) or (ontologies in Abstract near/6 enabler* in Abstract) or (ontologies in Abstract near/6 facilitator* in Abstract) or (ontologies in Abstract near/6 moderator* in Abstract) or (ontologies in Abstract near/6 mediator* in Abstract) or (ontologies in Abstract near/6 driver* in Abstract) or (ontologies in Abstract near/6 modifier* in Abstract) )

#28 ( (framework* in Record Title near/6 barrier* in Record Title) or (framework* in Record Title near/6 obstacle* in Record Title) or (framework* in Record Title near/6 impediment* in Record Title) or (framework* in Record Title near/6 hinder* in Record Title) or (framework* in Record Title near/6 enabler* in Record Title) or (framework* in Record Title near/6 facilitator* in Record Title) or (framework* in Record Title near/6 moderator* in Record Title) or (framework* in Record Title near/6 mediator* in Record Title) or (framework* in Record Title near/6 driver* in Record Title) or (framework* in Record Title near/6 modifier* in Record Title) )

#29 ( (framework* in Abstract near/6 barrier* in Abstract) or (framework* in Abstract near/6 obstacle* in Abstract) or (framework* in Abstract near/6 impediment* in Abstract) or (framework* in Abstract near/6 hinder* in Abstract) or (framework* in Abstract near/6 enabler* in Abstract) or (framework* in Abstract near/6 facilitator* in Abstract) or (framework* in Abstract near/6 moderator* in Abstract) or (framework* in Abstract near/6 mediator* in Abstract) or (framework* in Abstract near/6 driver* in Abstract) or (framework* in Abstract near/6 modifier* in Abstract) )

#30 ( ("frame work*" in Record Title near/6 barrier* in Record Title) or ("frame work*" in Record Title near/6 obstacle* in Record Title) or ("frame work*" in Record Title near/6 impediment* in Record Title) or ("frame work*" in Record Title near/6 hinder* in Record Title) or ("frame work*" in Record Title near/6 enabler* in Record Title) or ("frame work*" in Record Title near/6 facilitator* in Record Title) or ("frame work*" in Record Title near/6 moderator* in Record Title) or ("frame work*"

in Record Title near/6 mediator* in Record Title) or ("frame work*" in Record Title near/6 driver* in Record Title) or ("frame work*" in Record Title near/6 modifier* in Record Title) )

#31 ( ("frame work*" in Abstract near/6 barrier* in Abstract) or ("frame work*" in Abstract near/6 obstacle* in Abstract) or ("frame work*" in Abstract near/6 impediment* in Abstract) or ("frame work*" in Abstract near/6 hinder* in Abstract) or ("frame work*" in Abstract near/6 enabler* in Abstract) or ("frame work*" in Abstract near/6 facilitator* in Abstract) or ("frame work*" in Abstract near/6 moderator* in Abstract) or ("frame work*" in Abstract near/6 mediator* in Abstract) or ("frame work*" in Abstract near/6 driver* in Abstract) or ("frame work*" in Abstract near/6 modifier* in Abstract) )

#32 ( (classify in Record Title near/6 barrier* in Record Title) or (classify in Record Title near/6 obstacle* in Record Title) or (classify in Record Title near/6 impediment* in Record Title) or (classify in Record Title near/6 hinder* in Record Title) or (classify in Record Title near/6 enabler* in Record Title) or (classify in Record Title near/6 facilitator* in Record Title) or (classify in Record Title near/6 moderator* in Record Title) or (classify in Record Title near/6 mediator* in Record Title) or (classify in Record Title near/6 driver* in Record Title) or (classify in Record Title near/6 modifier* in Record Title) )

#33 ( (classify in Abstract near/6 barrier* in Abstract) or (classify in Abstract near/6 obstacle* in Abstract) or (classify in Abstract near/6 impediment* in Abstract) or (classify in Abstract near/6 hinder* in Abstract) or (classify in Abstract near/6 enabler* in Abstract) or (classify in Abstract near/6 facilitator* in Abstract) or (classify in Abstract near/6 moderator* in Abstract) or (classify in Abstract near/6 mediator* in Abstract) or (classify in Abstract near/6 driver* in Abstract) or (classify in Abstract near/6 modifier* in Abstract) )

#34 ( (classification* in Record Title near/6 barrier* in Record Title) or (classification* in Record Title near/6 obstacle* in Record Title) or (classification* in Record Title near/6 impediment* in Record Title) or (classification* in Record Title near/6 hinder* in Record Title) or (classification* in Record Title near/6 enabler* in Record Title) or (classification* in Record Title near/6 facilitator* in Record Title) or (classification* in Record Title near/6 moderator* in Record Title) or (classification* in Record Title near/6 mediator* in Record Title) or (classification* in Record Title near/6 driver* in Record Title) or (classification* in Record Title near/6 modifier* in Record Title) )

#35 ( (classification* in Abstract near/6 barrier* in Abstract) or (classification* in Abstract near/6 obstacle* in Abstract) or (classification* in Abstract near/6 impediment* in Abstract) or (classification* in Abstract near/6 hinder* in Abstract) or (classification* in Abstract near/6 enabler* in Abstract) or (classification* in Abstract near/6 facilitator* in Abstract) or (classification* in Abstract near/6 moderator* in Abstract) or (classification* in Abstract near/6 mediator* in Abstract) or (classification* in Abstract near/6 driver* in Abstract) or (classification* in Abstract near/6 modifier* in Abstract) )

#36 ( (theory in Record Title near/6 barrier* in Record Title) or (theory in Record Title near/6 obstacle* in Record Title) or (theory in Record Title near/6 impediment* in Record Title) or (theory in Record Title near/6 hinder* in Record Title) or (theory in Record Title near/6 enabler* in Record Title) or (theory in Record Title near/6 facilitator* in Record Title) or (theory in Record Title near/6 moderator* in Record

Title) or (theory in Record Title near/6 mediator* in Record Title) or (theory in Record Title near/6 driver* in Record Title) or (theory in Record Title near/6 modifier* in Record Title) )

#37 ( (theory in Abstract near/6 barrier* in Abstract) or (theory in Abstract near/6 obstacle* in Abstract) or (theory in Abstract near/6 impediment* in Abstract) or (theory in Abstract near/6 hinder* in Abstract) or (theory in Abstract near/6 enabler* in Abstract) or (theory in Abstract near/6 facilitator* in Abstract) or (theory in Abstract near/6 moderator* in Abstract) or (theory in Abstract near/6 mediator* in Abstract) or (theory in Abstract near/6 driver* in Abstract) or (theory in Abstract near/6 modifier* in Abstract) )

#38 ( (theories in Record Title near/6 barrier* in Record Title) or (theories in Record Title near/6 obstacle* in Record Title) or (theories in Record Title near/6 impediment* in Record Title) or (theories in Record Title near/6 hinder* in Record Title) or (theories in Record Title near/6 enabler* in Record Title) or (theories in Record Title near/6 facilitator* in Record Title) or (theories in Record Title near/6 moderator* in Record Title) or (theories in Record Title near/6 mediator* in Record Title) or (theories in Record Title near/6 driver* in Record Title) or (theories in Record Title near/6 modifier* in Record Title) )

#39 ( (theories in Abstract near/6 barrier* in Abstract) or (theories in Abstract near/6 obstacle* in Abstract) or (theories in Abstract near/6 impediment* in Abstract) or (theories in Abstract near/6 hinder* in Abstract) or (theories in Abstract near/6 enabler* in Abstract) or (theories in Abstract near/6 facilitator* in Abstract) or (theories in Abstract near/6 moderator* in Abstract) or (theories in Abstract near/6 mediator* in Abstract) or (theories in Abstract near/6 driver* in Abstract) or (theories in Abstract near/6 modifier* in Abstract) )

#40 (#22 or #23 or #24 or #25 or #26 or #27 or #28 or #29 or #30 or #31 or #32 or #33 or #34 or #35 or #36 or #37 or #38 or #39)

#41 MeSH descriptor communication barriers this term only

#42 (#21 or #41)

#43 (#40 or #42)

#44 sr-epoc in All Text

#45 (#43 and #44)

**Sociological Abstracts**

((((DE="Constraints") or(KW=((barrier* or constraint* or obstacle* or impediment* or hinder* or enabler* or facilitator* or moderator* or mediator* or driver* or modifier*) within 3 (identif* or elicit* or classif* or understand* or determin* or address* or examin* or study or "pick up" or "look up" or "weed out" or observ* or consider* or discover*)))) and(KW=(method* or model* or technique* or strategy or strategies or interview* or focus group* or questionnaire* or questioning or data collection or client* record* or medical record* or dental record* or hospital record* or nursing record* or patient* record* or brainstorming or brain storming or informant* or discuss* or direct observation or participant observation or informal observation or survey* or nominal group* or qualitative research or grounded theory or qualitative study or qualitative studies or case study or case studies or case

report* or anthropology or ethnology or ethnography or phenomenology or narrative* or narration*))) and((KW=((quality) within 3 (improv* or health care or healthcare or medical care or dental care or nursing care))) or(KW=((improv* or influenc*) within 3 (health care or healthcare or medical care or dental care or nursing care or practice* or competence or prescri*))) or(KW=((clinical guideline* or practice guideline* or clinical path* or critical path*) within 3 (implement* or adher* or comply or compliance))) or(KW=evidence based) or(KW=((tailor* or develop* or link*) within 3 (intervention*))) or(KW=(knowledge treasfer* or knowledge translat*)))) or(KW=((taxonomy or taxonomies or ontology or ontologies or framework* or frame work* or classify or classification* or theory or theories) within 3 (barrier* or constraint* or obstacle* or impediment* or hinder* or enabler* or facilitator* or moderator* or mediator* or driver* or modifier*)))

**Science Citation Index & Social Sciences Citation Index**

**Methods:**
Title=((barrier* or enabler* or facilitator* or moderator* or mediator* or modifier*)) AND Topic=((method* or model* or technique* or strateg* or interview* or "focus group" or "focus groups" or questionnaire* or brainstorming or "brain storming" or informant* or survey* or "nominal group" or "nominal groups" or "qualitative research" or "qualitative study" or "qualitative studies" or "grounded theory" or "case study" or "case studies" or "case report" or "case reports")) AND Topic=(("quality improvement" or implement* or (guideline* and adhere*) or (path* and adhere*) or (tailor* and intervention*) or "evidence based" or "knowledge transfer" or "transfering knowledge" or "transferring knowledge" or "knowledge translation")) AND Topic=((health* or medical))

**Taxonomies**:
Title=((barrier* or enabler* or facilitator* or moderator* or mediator* or modifier*)) AND Topic=((taxonomy or taxonomies or framework* or "frame work" or "frame works" or classification*)) AND Topic=((health* or medical))
