# Supplementary material for: A checklist for identifying determinants of practice: A systematic review and synthesis of frameworks and taxonomies of factors that prevent or enable improvements in healthcare professional practice
Source: Implement Sci. 2013 Mar 23;8:35. doi: 10.1186/1748-5908-8-35 (PMC3617095; doi:10.1186/1748-5908-8-35)
Supplement: Additional file 2 — Feedback on TICD Checklist. [file 1748-5908-8-35-S2.pdf]

Additional file 2 - Feedback on TICD Checklist

Name(s):

Date:

				Comments (including explanations of perceived problems and suggestions for improvements)
**Comprehensiveness**<br>**1.** Are potentially important factors missing from the checklist?	Yes ☐	Uncertain ☐	No ☐	
**Relevance**<br>**2.** Are factors included in the checklist that should not be?	No ☐	Uncertain ☐	Yes ☐	
**Applicability**<br>**3.** Is checklist applicable across different settings (e.g. primary and secondary care) and different types of practices (including prevention, diagnosis and treatment for chronic and non-chronic conditions)?	Yes ☐	Uncertain ☐	No ☐	
**Simplicity**<br>**4.** Is the checklist more complicated than necessary?	No ☐	Uncertain ☐	Yes ☐	
**Logic**<br>**5.** Is the checklist organised in a logical way that is easy to understand?	Yes ☐	Uncertain ☐	No ☐	
**Clarity**<br>**6.** Are the factors and domains (groups of factors) labelled and explained in a way that is easy to understand?	Yes ☐	Uncertain ☐	No ☐	
**Usability**<br>**7.** Would it be easy for implementation researchers to use the checklist and the worksheets?	Yes ☐	Uncertain ☐	No ☐	
**8.** Would it be easy for people who are not implementation researchers to use the checklist and worksheets?	Yes ☐	Uncertain ☐	No ☐	
**Suitability**<br>**9.** Are the checklist and worksheets suitable for helping people to identify and prioritise determinants of change in practice that should be considered when designing implementation strategies?	Yes ☐	Uncertain ☐	No ☐	
**Usefulness**<br>**10.** Are the checklist and worksheets likely to be useful to people designing implementation strategies?	Yes ☐	Uncertain ☐	No ☐	
**11.** Is the checklist likely to be useful for reporting determinants of practice in research reports?	Yes ☐	Uncertain ☐	No ☐	

		Comments (including explanations of perceived problems and suggestions for improvements)
**Overall assessment**<br>**12.** Overall, are the checklist and worksheets adequate to be used to identify and prioritise determinants of practice?	Yes ☐ Partially ☐ No ☐<br>See explanation below.	
**Strengths**<br>**13.** What do you like about the checklist and worksheets?		
**Weaknesses**<br>**14.** What don't you like about the checklist and worksheets and what suggestions do you have for improving them?		
**Anything else**<br>**15.** Please include any other comments you have regarding the checklist or worksheets.		

Overall assessment
Yes = Could be used as is with little or no modification
Partially = Needs some modification or further development
No = Not adequate
