# Supplementary material for: A checklist for identifying determinants of practice: A systematic review and synthesis of frameworks and taxonomies of factors that prevent or enable improvements in healthcare professional practice
Source: Implement Sci. 2013 Mar 23;8:35. doi: 10.1186/1748-5908-8-35 (PMC3617095; doi:10.1186/1748-5908-8-35)
Supplement: Additional file 3 — Dimensions and factors from 12 comprehensive checklists. [file 1748-5908-8-35-S3.pdf]

# Additional file 3 Dimensions and factors from 12 comprehensive checklists

## Cabana 1999 [1]

Knowledge:
**Lack of familiarity** - volume of information, time needed to stay informed, guideline accessibility
**Lack of awareness** - Volume of information, time needed to stay informed, guideline accessibility
Attitudes:
**Lack of agreement with specific guidelines** - interpretation of evidence, applicability to patient, not cost-beneficial, lack of confidence in guideline developer
**Lack of agreement with guidelines in general** - too cookbook, too rigid to apply, biased synthesis, challenge to autonomy, not practical
**Lack of outcome expectancy** - physician believes that performance of guideline recommendations will not lead to desired outcomes
**Lack of self-efficacy** - physician believes that he/she cannot perform guideline recommendation
**Lack of motivation/inertia of previous practice** - habit, routines
Behaviour:
**External barriers** - patient factors (inability to reconcile patient preferences with guideline recommendations
**Guideline factors** - guideline characteristics, presence of contradictory guidelines
**Environmental factors** - lack of time, lack of resources, organizational constraints, lack of reimbursement, perceived increase in malpractice liability

## Cochrane 2007 [2]

**Cognitive/behavioural barriers** - knowledge, awareness, skill/expertise, critical appraisal skills
**Attitudinal/rational-emotive barriers** - efficacy/perceived competence, perceived/outcome expectancy, confidence in abilities, authority, accurate self-assessment
**Health care professional/physician barriers** - characteristics, age/maturity of practice, professional boundaries, legal issues, peer influence/models, gender, inertia
**Clinical practice guidelines/evidence barriers** - utility, evidence/disagree content, access, structure, local applicability
**Patient barriers** - patient characteristics/factors, patient adherence
**Support/resources barriers** - time, support, costs/funding issues, resources
**System/process barriers** - organizational, system, HR/workload/overload, team structure/work, referral process

## Damschroder 2009 [3]

**Intervention characteristics** - intervention source, evidence strength & quality, relative advantage, adaptability, trialability, complexity, design quality and packaging, cost
**Outer setting** - patient needs & resources, cosmopolitanism, peer pressure, external policies & incentives
**Inner setting** - structural characteristics, networks & communications, culture, implementation climate (tension for change, compatibility, relative priority, organizational incentives & rewards, goals and feedback, learning climate), readiness for implementation (leadership engagement, available resources, access to knowledge and information)
**Characteristics of individuals** - knowledge & beliefs about the intervention, self-efficacy, individual stage of change, individual identification with organization, other personal attributes
**Process** - planning, engaging (opinion leaders, formally appointed internal implementation leaders, champions, external change agents), executing, reflecting & evaluating

## Greenhalgh 2004 [4]

**Innovation** - inherent attributes (relative advantage, compatibility, low complexity, trialability, observability, reinvention), operational attributes (task relevance, task usefulness, feasibility, implementation complexity, divisibility, nature of knowledge needed)
**Adopters and adoption** - characteristics and needs, meaning of the innovation, nature of the adoption decision, concerns (at pre-adoption stage, early use stage, experienced user stage)
**Communication and influence** - nature of networks, main agents of social influence
**Inner context** - structural features of the organisation (size/maturity, complexity/differentiation, decentralisation, slack resources), organisation's absorptive capacity for new knowledge (skill mix, knowledge base, transferable know-how, ability to evaluate the innovation), receptive context (leadership and vision, values and goals, risk-taking climate, internal and external networks), organisation's readiness (organisational fit, assessment of implications, dedicated time/resources, broad based support)
**Outer context** - socio-political climate, external incentives and mandates, prevailing norms from other comparable ('opinion leader') organisations

**Implementation and sustainability** - implementation process (human resources, involvement of key staff, project management), response to consequences of innovation (e.g. audit and feedback), measures to adapt and re-invent the innovation (e.g. inter-organizational networks and collaboratives)
**External agencies** - linkage between developers and users of the innovation at the development stage, shared value systems, shared language and meanings, external change agents (homophily, positive relationships and client-centeredness, shared language and meaning), social marketing principles (audience segmentation, assessment of target group needs and perspective, appropriate message and marketing channels, good programme management, process evaluation), relationship between change agency and intended adopter organisations

## Gurses 2010 [5]

**Clinician characteristics** - awareness, familiarity, agreement, self-efficacy, outcome expectancy, motivation/inertia of previous practice, normative beliefs, subjective norm
**Guideline characteristics** - relative advantage, compatibility, complexity, trialability, observability, strength of evidence, exception ambiguity
**System characteristics** - task characteristics (e.g. workload, task ambiguity, responsibility ambiguity, method ambiguity), tools-technologies (e.g. supplies, checklist, method ambiguity), physical environment (e.g. layout, workspace, noise), organizational characteristics (e.g. culture, teamwork, communication, expectation ambiguity, method ambiguity)
**Implementation characteristics** - tension for change, mandate/preparation-planning, leader and middle manager involvement and support, change agents' characteristics, relative strength of supporters (opinion leaders) and opponents, exploration of problem and customer/staff needs, getting ideas from outside the organization, funding availability, monitoring and feedback mechanisms, clarity and simplicity of the implementation plan, segmentation and targeting, exchange of value and marketing mix (4 Ps), attention to behavioural competition

## Kitson 2008 [6]

**Evidence** - research, clinical experience, patient experience
**Context** - context, culture, leadership, evaluation
**Facilitation** - purpose, role, skills and attributes

## Mäkelä 1999 [7]

**Professionals**:
**Knowledge** - knowledge about existence of guideline, knowledge about own practice differing from guideline, knowledge for complying with guideline, other (specify)
**Skills** - skills for locating or fetching guideline (e.g. from Internet), individual skills for complying with guideline, team skills for complying with guideline, organisational competence for complying with the guideline, other (specify)
**Attitudes** - patient's values differ from those of the professional or those in the guideline, relative's values differ from those of the professional or those in the guideline, social group's values differ from those of the professional or those in the guideline, other (specify)
**Other resources** - money, assistance, other (specify)
**Patients:**
**Knowledge -** knowledge about existence of guideline, understanding of guideline content, other (specify)
**Skills** - understanding recommendations by the professional, ability to follow recommendations by the professional, other (specify)
**Attitudes** - patient’s values differ from those of the professional, or those in the guideline, relative’s values differ from those of the professional or those in the guideline, social group’s values differ from those of the professional or those in the guideline, other (specify)
**Other resources** – money, assistance, other (specify)
**Environment**:
**Social factors** - support for or discouragement of change by others (colleagues at practice site, other members of professional team, managers, other local health care providers, opinion leaders, patients, professional organisations, patient organisations, other (specify)
**Organisational factors** - availability of guidelines at workplace, practicality within existing practice setting or routines, local infrastructures or rules, other (specify)
**Economic factors** - availability/lack of resources (time, personnel etc.), change in income for provider, changed cost for patient, changed cost for practice organisation, changed cost for health care system, other (specify)

## Michie 2005 [8]

**Knowledge** - knowledge, knowledge about condition / scientific rationale, schemas+mindsets+illness representations, procedural knowledge
**Skills** - skills, competence/ability/skill assessment, practice / skills development, interpersonal skills, coping strategies
**Social/professional role and identity (self-standards)** - identity, professional identity/boundaries/role, group/social identity,

social/group norms, alienation/organisational commitment
**Beliefs about capabilities (self-efficacy)** - self-efficacy, control-of behaviour and material and social environment, perceived competence, self-confidence/professional confidence, empowerment, self-esteem, perceived behavioural control, optimism/pessimism
**Beliefs about consequences (anticipated outcomes/attitude)** - outcome expectancies, anticipated regret, appraisal/evaluation/review, consequents, attitudes, contingencies, reinforcement/punishment/consequences, incentives/rewards, beliefs, unrealistic optimism, salient events/sensitisation/critical incidents, characteristics of outcome expectancies (physical, social, emotional), sanctions/rewards (proximal/distal, valued/not valued, probable/improbable, salient/not salient, perceived risk/threat)
**Motivation and goals (intention)** - intention (stability of intention/certainty of intention), goals (autonomous, controlled), goal target/setting, goal priority, intrinsic motivation, commitment, distal and proximal goals, transtheoretical model and stages of change
**Memory, attention and decision processes** - memory, attention, attention control, decision making
**Environmental context and resources (environmental constraints)** - resources/material resources (availability and management), environmental stressors, person x environment interaction, knowledge of task environment
**Social influences (norms)** - social support, social/group norms, organisational development, leadership, team working, group conformity, organisational climate/culture, social pressure, power/hierarchy, professional boundaries/roles, management commitment, supervision, inter-group conflict, champions, social comparisons, identity (group/social identity), organisational commitment/alienation, feedback, conflict (competing demands, conflicting roles, change management, crew resources management, negotiation, social support (personal/professional/organisational, intra/interpersonal, society/community)
**Emotion** - affect, stress, anticipated regret, fear, burn-out, cognitive overload/tiredness, threat, positive/negative affect, anxiety/depression
**Behavioural regulation** - goal/target setting, implementation intention, action planning, self-monitoring, goal priority, generating alternatives, feedback, moderators of intention-behaviour gap, project management, barriers and facilitators
**Nature of the behaviours** - routine/automatic/habit, breaking habit, direct experience/past behaviour, representation of tasks, stages of change model

## NICS 2006 [9]

**The innovation itself** - feasibility, credibility, accessibility, attractiveness
**Individual professional** - awareness, knowledge, attitude, motivation to change, behavioural routines
**Patient** - knowledge, skills, attitude, compliance
**Social context** - opinion of colleagues, culture of the network, collaboration, leadership
**Organisational context** - care processes, staff, capacities, resources, structures
**Economic and political context** - financial arrangements, regulations, policies

## Saillour-Glenisson 2003 [10]

**Clinical practice guideline (CPG) characteristics** - form, compatibility, trialability, scientific basis, observability, adaptability, legal implications
**Physician characteristics** - knowledge about the CPG, attitude and agreement to CPG, psychological and socio-demographic and economic characteristics, job satisfaction, training
**Physician environment** - physician human environment (patient influence, peer influence), physician organizational environment (internal environment, external environment)

## SURE 2011 [11]

**Recipients of care** - knowledge and skills, attitudes regarding programme acceptability, appropriateness and credibility, motivation to change or adopt new behaviour
**Providers of care** - knowledge and skills, attitudes regarding programme acceptability, appropriateness and credibility, motivation to change or adopt new behaviour
**Other stakeholders** (including other healthcare providers, community health committees, community leaders, programme managers, donors, policy makers and opinion leaders)- knowledge and skills, attitudes regarding programme acceptability, appropriateness and credibility, motivation to change or adopt new behaviour
**Health system constraints** - accessibility of care, financial resources, human resources, educational system, clinical supervision, internal communication, external communication, allocation of authority, accountability, management or leadership, information systems, facilities, patient flow processes, procurement and distribution systems, incentives, bureaucracy, relationship with norms and standards
**Social and political constraints** - ideology, short-term thinking, contracts, legislation or regulations, donor policies, influential people, corruption, political stability

## Wensing 2005 [12]

<u>**Individual level:**</u>
**Cognitive factors** - information behaviour, domain knowledge
**Motivational factors** - motivation, beliefs about consequences, attitudes, perceived subjective norms, beliefs about capabilities, emotion
**Behavioural factors** - behavioural regulation, skills
<u>**Professional interaction:**</u>
**Interaction in professional teams** - team cognitions, team processes
**Structure of professional networks** - leadership and key individuals, social network characteristics
<u>**Organisational level:**</u>
**Organizational structures** - specification, flexibility, leadership structure, specialization
**Organizational processes** - continuous improvement, external communication, internal communication
**Organizational resources** - technical knowledge, organizational size
<u>**Factors related to structures:**</u>
**Societal factors** - professional development, priority on societal agenda
**Financial incentives** - positive incentives, provider and patient financial risk sharing, transaction costs, competition intensity
**Regulations** - purchaser-provider contract relationships

## References


1. Cabana MD, Rand CS, Powe NR, Wu AW, Wilson MH, Abboud PA *et al.*: **Why don't physicians follow clinical practice guidelines? A framework for improvement.** *JAMA* 1999, **282:** 1458-1465.
2. Cochrane LJ, Olson CA, Murray S, Dupuis M, Tooman T, Hayes S: **Gaps between knowing and doing: Understanding and assessing the barriers to optimal health care.** *J Contin Educ Health Prof* 2007, **27:** 94-102.
3. Damschroder LJ, Aron DC, Keith RE, Kirsh SR, Alexander JA, Lowery JC: **Fostering implementation of health services research findings into practice: a consolidated framework for advancing implementation science.** *Implement Sci* 2009, **4:** 50.
4. Greenhalgh T, Robert G, Bate P, Kyriakidou O, Macfarlane F, Peacock R. **How to spread good ideas: A systematic review of the literature on diffusion, dissemination and sustainability of innovations in health service delivery and organisation.** *Report for the National Co-ordinating Centre for NHS Service Delivery and Organisation R & D (NCCSDO)*. London: NCCSDO; 2004.
5. Gurses AP, Marsteller JA, Ozok AA, Xiao Y, Owens S, Pronovost PJ: **Using an interdisciplinary approach to identify factors that affect clinicians' compliance with evidence-based guidelines.** *Crit Care Med* 2010, **38:** S282-S291.
6. Kitson AL, Rycroft-Malone J, Harvey G, McCormack B, Seers K, Titchen A: **Evaluating the successful implementation of evidence into practice using the PARiHS framework: theoretical and practical challenges.** *Implement Sci* 2008, **3:** 1.
7. Mäkelä M, Thorsen T: **A framework for guidelines implementation studies.** In *Changing professional practice. Theory and practice of clinical guidelines implementation*. First edition. Edited by Thorsen T, Mäkelä M. Copenhagen: DSI - Danish Institute for Health Services Research; 1999:23-53.
8. Michie S, Johnston M, Abraham C, Lawton R, Parker D, Walker A: **Making psychological theory useful for implementing evidence based practice: a consensus approach.** *Qual Saf Health Care* 2005, **14:** 26-33.
9. Rainbird K, Sanson-Fisher R, Buchan H. **Identifying barriers to evidence uptake.** Melbourne: National Institute of Clinical Studies (NICS); 2006.
10. Saillour-Glenisson F, Michel P: **[Individual and collective facilitators of and barriers to the use of clinical practice guidelines by physicians: a literature review].** *Rev Epidemiol Sante Publique* 2003, **51:** 65-80.
11. SURE Collaboration. **SURE Guides for Preparing and Using Evidence-Based Policy Briefs: 5. Identifying and addressing barriers to implementing policy options.** Version 2.0 [updated August 2011]. The SURE Collaboration, 2011.

12. Wensing M, Bosch M, Foy R, van der Weijden T, Eccles M, Grol R. **Factors in theories on behaviour change to guide implementation and quality improvement in health care.** Nijmegen: Centre for Quality of Care Research (WOK); 2005.
