# Supplementary material for: A checklist for identifying determinants of practice: A systematic review and synthesis of frameworks and taxonomies of factors that prevent or enable improvements in healthcare professional practice
Source: Implement Sci. 2013 Mar 23;8:35. doi: 10.1186/1748-5908-8-35 (PMC3617095; doi:10.1186/1748-5908-8-35)
Supplement: Additional file 4 — TICD Checklist – definitions, questions and examples. [file 1748-5908-8-35-S4.pdf]

Additional file 4 TICD Checklist – definitions, questions an and examples

Determinants	Definitions	Questions	Examples of specific factors[1]	Examples of related implementation strategies	Methods for identifying the determinants
**1. GUIDELINE FACTORS**					
• **Recommendation**					
• **Quality of evidence supporting the recommendation**	How confident we are in the estimates of effects	What is the quality of the evidence supporting the recommendation and has it been assessed appropriately?	The quality of the evidence that supports the recommendation may not be clear or may not be judged appropriately	Clearly and accurately communicate the quality of the evidence; Don't invest resources in implementing recommendations for which there is low quality evidence	Critical review of the guideline using GRADE[i]
• **Strength of recommendation**	How confident we are that the desirable effects of adherence to the recommendation outweigh the undesirable effects	What is the strength of the recommendation, has it been assessed appropriately, and are the implications of the strength of the recommendation clearly communicated?	The strength of the recommendation may not be clear or appropriate, or the implications of a weak recommendation may not be clearly communicated[2]	Clearly communicate the implications of the strength of the recommendation	Critical review of the guideline using GRADE[ii]
• **Clarity**	The clearness of the target population, the settings in which the recommendation is to be used and the recommended action	Is the recommended action (what to do) stated specifically and unambiguously? Is sufficient detail provided to allow the targeted healthcare professionals to perform the recommended action?	The recommendation may be ambiguous, lack sufficient detail or be longwinded	Clearly communicate a specific and unambiguous action with sufficient detail about how to do it to allow the targeted healthcare professionals to perform the recommended action	Critical review of the guideline using the GuideLine Implementability Appraisal (GLIA)[iii]
• **Cultural appropriateness**	The extent to which the recommendation is suitable in the social context where it is being implemented	Is the recommendation culturally appropriate?	The recommendation may not be congruous with customs or norms in the context where they are being implemented	Adapt the recommendation so that it is congruous; Communicate the recommendation in a way that is more congruous	Reflexion; Interviews or focus group discussion with targeted healthcare professionals and with patients

[1] The examples are presented as barriers that might hinder implementation of a recommendation. However, factors can also befacilitators that could be capitalised on to help implement a recommendation.

[2] I.e. it may be implied that a weak recommendation is not important rather than that clinicians should be prepared to help patients to make decisions that are consistent with their own values.

Determinants	Definitions	Questions	Examples of specific factors[1]	Examples of related implementation strategies	Methods for identifying the determinants
• **Accessibility of the recommendation**	How accessible the guideline or recommendation is	Is the guideline or recommendation accessible	The guideline may be long or poorly written; The recommendation may be buried in the guideline or one of a long list of recommendations; The guideline may not be available in a format (e.g. electronically) that appeals to the targeted healthcare professionals	Provide a concise guideline with easily identifiable recommendations in a format that appeals to the targeted healthcare professionals	Critical review of the guideline using the GuideLine Implementability Appraisal (GLIA) [iii]
• **Source of the recommendation**	The organisation(s) and people that made the recommendation	Do the organisation(s) and people who made the recommendation have credibility with the targeted healthcare professionals?	The organisation or people that made the recommendation may lack credibility with the targeted healthcare professionals	Ensure that the recommendation is made or endorsed by an organisation and people that have credibility with the targeted healthcare professionals	Ask targeted healthcare professionals about their perceptions of the credibility of the organisation and people who made the recommendation
• **Consistency with other guidelines**	The extent to which the recommendation is consistent with recommendations in other guidelines with which the targeted healthcare professionals might be familiar	Is the recommendation consistent with recommendations in other guidelines with which the targeted healthcare professionals might be familiar?	Conflicting recommendations may be confusing	Explain the reasons for conflicting recommendations to the targeted healthcare professionals	Examine the reasons for conflicting recommendations [iv]
**Recommended clinical intervention**					
• **Feasibility**	The extent to which the recommended clinical intervention is practical	Is the recommended clinical intervention feasible for the targeted healthcare professionals?	The recommended clinical intervention may not be practical in some settings or may be perceived as not being practical	Ensure that the clinical intervention is practical; Provide necessary assistance to make it more practical; Address misperceptions of its practicality	Ask targeted healthcare professionals; Pilot test use of the clinical intervention in the targeted settings
• **Accessibility of the intervention**	The extent to which the recommended clinical intervention is accessible	Is the recommended clinical intervention accessible?	The recommended clinical intervention may not be accessible in some settings	Ensure that the clinical intervention is accessible; Make it more accessible	Routinely collected data; Survey people in the targeted settings
**Recommended behaviour**					
• **Compatibility**	The extent to which the recommended behaviour fits with current practices	Is the recommended behaviour compatible with existing practices?	The recommendation may disrupt the current work flow	Information or education that helps the targeted healthcare professionals to fit the recommended behaviour into their current practice	Focus group discussion with targeted healthcare professionals
• **Effort**	The amount of effort required to change or adhere	How much effort is required to adhere?	Adherence may require a substantial effort	Reduce the effort required, provide assistance or provide motivation	Interviews or focus group discussion with targeted healthcare professionals

Determinants	Definitions	Questions	Examples of specific factors[1]	Examples of related implementation strategies	Methods for identifying the determinants
• **Trialability**	The ability to try out the recommended behaviour	Is it possible to try out the recommended behaviour?	The targeted healthcare professionals may be reluctant to change their behaviour if they cannot first try it out in conducive circumstances	Provide the targeted healthcare professionals the opportunity to try out the behaviour; e.g. at an educational meeting or with a simulated patient	Interviews or focus group discussion with targeted healthcare professionals
• **Observability**	The degree to which benefits of the recommended behaviour are visible	Are the benefits of adhering to the recommendation observable?	The targeted healthcare professionals may have doubts about the benefits of adhering to the recommendation	Provide feedback, information or education regarding the benefits of adhering to the recommendation	Interviews or focus group discussion with targeted healthcare professionals about their perceptions of indicators of adherence or outcomes that can be measured
**2. INDIVIDUAL HEALTH PROFESSIONAL FACTORS**					
• **Knowledge and skills**					
• **Domain knowledge**	The extent to which the targeted healthcare professionals have pre-existing knowledge or expertise about the targeted condition	What knowledge or expertise could affect how the targeted healthcare professionals learn what they need to know to adhere and to what extent do they have that knowledge or expertise?	Experts and non-experts may have different educational needs; Implementing a recommendation based on new knowledge may require targeted efforts to disseminate that new knowledge	Change the mix of professional skills in the targeted teams or organisations; Tailor educational strategies to the level of expertise of the targeted healthcare professionals; Targeted dissemination of new knowledge	Routinely collected data regarding human resources for health; Interviews or focus group discussion with targeted healthcare professionals
• **Awareness and familiarity with the recommendation**	The extent to which the targeted healthcare professionals are aware of and familiar with the recommendation	Are the targeted healthcare professionals aware of and familiar with the recommendation?	The volume of information, time needed to stay informed, and guideline accessibility	A dissemination strategy that is tailored to reach the targeted healthcare professionals	Survey, interviews or focus group discussion with targeted healthcare professionals
• **Knowledge about own practice**	The extent to which the targeted healthcare professionals are aware of their own practice in relationship to the recommended practice	Are the targeted healthcare professionals aware of the extent to which they are adhering to the recommendation?	The targeted healthcare professionals may not be aware of their own performance	Audit and feedback	Audit and survey, interviews or focus group discussion with targeted healthcare professionals
• **Skills needed to adhere**	The extent to which the targeted health professionals have skills that they need to adhere	What skills are needed to adhere and do the targeted health professionals have those?	The targeted health professionals may not have the necessary skills	Educational strategies with opportunities to practice necessary skills	Interviews or focus group discussion with the targeted health professionals

Determinants	Definitions	Questions	Examples of specific factors[1]	Examples of related implementation strategies	Methods for identifying the determinants
**Cognitions (including attitudes)**					
• **Agreement with the recommendation**	The extent to which the targeted healthcare professionals agree with the recommendation	Do the targeted healthcare professionals agree with the recommendation? If not, why?	The targeted healthcare professionals may interpret the quality of the evidence or its applicability differently, may not think the recommended intervention is cost-effective, or may lack of confidence in the guideline developer	Educational strategies that address the reasons for disagreement; A local consensus process	Survey, interviews or focus group discussion with targeted healthcare professionals
• **Attitudes towards guidelines in general**	The perceptions that the targeted healthcare professionals have regarding guidelines in general	How do the targeted healthcare professionals view guidelines in general?	The targeted healthcare professionals may perceive guidelines as being oversimplified or cookbook, lacking sufficient flexibility or adaptability, restricting their autonomy, or not practical	Use strategies that do not depend on the targeted healthcare professionals's attitudes towards guidelines; Educational strategies that address negative attitudes towards guidelines; Design guidelines to address legitimate concerns	Survey, interviews or focus group discussion with targeted healthcare professionals
• **Expected outcome**	The extent to which the targeted healthcare professionals believe that adherence with the recommendation will lead to desired outcomes	Do the targeted healthcare professionals believe that adherence with the recommendation will lead to desired outcomes?	The targeted healthcare professionals may not believe that adherence with the recommendation will lead to desired outcomes	Information or educational strategies that provide compelling evidence; Audit and feedback	Survey, interviews or focus group discussion with targeted healthcare professionals
• **Intention and motivation**	The extent to which the targeted healthcare professionals intend to adhere and are motivated to do so	Do the targeted healthcare professionals intend to adhere? Are they motivated to adhere? What concerns do they have about adhering to the recommendation?	The targeted healthcare professionals may not intend to adhere; They may not be persuaded to change their behaviour due to inertia or their stage of change[3]	Local discussion and consensus; Discuss resistance; Provide good arguments why adherence is important; Involve opinion leaders; Strategies that are tailored to the stage of change of individuals in the targeted healthcare professional [v]	Survey, interviews or focus group discussion with targeted healthcare professionals
• **Self-efficacy**	The targeted healthcare professionals' self-perceived competence or confidence in their abilities	Do the targeted healthcare professionals believe that they are capable of adhering to the recommendation? If not, why?	The targeted healthcare professionals may lack confidence in their ability to adhere	Skills training; Feedback; Education or counselling to change the targeted healthcare professionals' self-assessment of their competency	Interviews or focus group discussion with targeted healthcare professionals

[3] Stages of change include contemplation, planning and undertaking actions, and appraising the outcomes of those actions. Individuals in different stages may be motivated by different factors.

Determinants	Definitions	Questions	Examples of specific factors[1]	Examples of related implementation strategies	Methods for identifying the determinants
• **Learning style**	The preferred ways in which the targeted healthcare professionals learn	What types of continuing education do the targeted healthcare professionals prefer?	Continuing education strategies that do not fit with the preferences or routines of the targeted healthcare professionals may not be effective	Selection of continuing education strategies that fit with the preferences and routines of the targeted healthcare professionals	Survey, interviews or focus group discussion with targeted healthcare professionals
• **Emotions**	The extent to which emotions affect adherence	Do the targeted healthcare professionals have emotions that facilitate or hinder adherence?	Adherence may be hindered by a lack of satisfaction, frustration, empathy or a lack of empathy, stress or burnout, cognitive overload or tiredness, or anticipated regret or fear	Strategies to increase satisfaction or reduce dissatisfaction; Strategies to reduce stress	Interviews or focus group discussion with targeted healthcare professionals
**Professional behaviour**					
• **Nature of the behaviour**	Characteristics of the behaviour, including: frequency of performance for a patient, frequency of performance for a population of patients, the degree of habit or automaticity, whether it is within a sequence of other behaviours that have to be performed, and whether it is performed by one person or by different people	What do people currently do? Who needs to do what differently when, where, how, how often and with whom?	The targeted healthcare professionals may need to break a habit, establish a new habit, or be prompted to do something that is not routine	Modification of the context to prompt the new behaviour; e.g. reminders, changes in layout or equipment	Interviews or focus group discussion with targeted healthcare professionals
• **Capacity to plan change**	The extent to which the targeted healthcare professionals have the capacity to plan necessary changes in order to adhere	What changes do the targeted healthcare professionals need to plan in order to adhere? Do they have the capacity to do this?	The targeted healthcare professionals may lack time or skills to plan necessary changes	Extra resources, support or aids to assist with planning necessary changes; Provide feasible objectives for change, an inventory of typical problems and solutions	Interviews or focus group discussion with targeted healthcare professionals
• **Self-monitoring or feedback**	The extent to which the targeted healthcare professionals have the capacity for self-monitoring or feedback to reinforce adherence with the recommendation	Is self-monitoring or feedback of adherence with the recommendation needed? If so, does the targeted healthcare professionals have the capacity for this?	The targeted healthcare professionals may relapse to previous behaviours or forget to adhere; They may lack necessary ongoing support or resources to maintain adherence	Monitoring, feedback, reminder systems; Integration of the recommendation into routine care plans or local protocols; Provide necessary resources, support or rewards	Interviews or focus group discussion with targeted healthcare professionals
**3. PATIENT FACTORS**					
• **Patient needs**	Real or perceived needs and demands of the patient	What are the targeted healthcare professionals' perceptions of the needs and demands of patients? How do those perceptions correspond with actual patient needs and demands?	Patients may make demands that hinder adherence; The targeted healthcare professionals may have misperceptions of patients needs or demands	Patient education materials; Provide the targeted healthcare professionals with accurate information about patient needs and demands	Interviews or focus group discussion with targeted healthcare professionals and with patients

Determinants	Definitions	Questions	Examples of specific factors[1]	Examples of related implementation strategies	Methods for identifying the determinants
• **Patient beliefs and knowledge**	Patients' beliefs or knowledge or ability to learn, or the targeted healthcare professionals' ability or perceived ability to inform or teach patients necessary knowledge and skills	What are the targeted healthcare professionals' perceptions of patients' knowledge or ability to learn, or the targeted healthcare professionals' ability or perceived ability to inform or teach patients necessary knowledge and skills? How do those perceptions correspond with actual patient knowledge and ability to learn?	Patients may have beliefs that hinder implementation of the recommendation; Patients may lack necessary knowledge or skills; The targeted healthcare professionals may lack confidence in their ability to adequately educate patients; The targeted healthcare professionals may have misperceptions of patients' beliefs, knowledge, skills or ability to learn	Patient education materials; Train the targeted healthcare professionals to provide patient education; Shift responsibility for patient education; Provide the targeted healthcare professionals with accurate information about patients knowledge, skills or ability to learn	Interviews or focus group discussion with targeted healthcare professionals and with patients
• **Patient preferences**	Patients' values in relationship to professional values or those in the recommendation	Do the targeted healthcare professionals perceive patients to have values that are different than their own or those in the recommendation? How do those perceptions correspond with actual patient values?	Patients may have values that are different than those of the targeted healthcare professionals or those in the recommendation; The targeted healthcare professionals may have misperceptions of patient values	Change the recommendation if it is based on values that are different from those of the targeted patients; A decision aid for patients to help them clarify their values; Provide the targeted healthcare professionals with accurate information about patient values	Interviews or focus group discussion with targeted healthcare professionals and with patients
• **Patient motivation**	The targeted healthcare professionals' ability or perceived ability to motivate patients to adhere	Do the targeted healthcare professionals perceive difficulties motivating patients to adhere? How do those perceptions correspond with actual patient motivation?	Patients may not be motivated to adhere; The targeted healthcare professionals may have misperceptions of patients' motivation	Provide the targeted healthcare professionals with aids or strategies to motivate patients; Shift responsibility for motivating patients; Provide the targeted healthcare professionals with accurate information about patients' motivation	Interviews or focus group discussion with targeted healthcare professionals and with patients
• **Patient behaviour**	Patient behaviours that motivate or demotivate adherence with the recommendation	Do the targeted healthcare professionals experience patients behaving in ways that discourage them from adhering?	Patients may behave in ways that hinder adherence (e.g. they may not adhere to recommended treatment or they may under or overuse health services)	Provide targeted healthcare professionals with strategies for coping with patient behaviours that are demotivating	Interviews or focus group discussion targeted healthcare professionals and with patients

Determinants	Definitions	Questions	Examples of specific factors[1]	Examples of related implementation strategies	Methods for identifying the determinants
**4. PROFESSIONAL INTERACTIONS**					
• **Communication and influence**	The extent to which the targeted healthcare professionals' adherence is influenced by professional opinions and communication	Is the targeted healthcare professionals' adherence influenced by professional organisations, professional networks, prevailing norms (opinions of colleagues) or opinion leaders (or champions or other influential people)?	Opinions and communication among professionals may hinder adherence	Engage professional organisations in guideline development or a consensus process; Obtain endorsement of the recommendation by professional organisations or opinion leaders; Identify champions to promote implementation of the recommendation	Interviews or focus group discussion with targeted healthcare professionals
• **Team processes**	The extent to which professional teams or groups have the skills needed to adhere and interact in ways that facilitate or hinder adherence	What skills do targeted professional teams or groups need to have? Do they have those skills? Do they interact in ways that facilitate or hinder adherence with the recommendation?	Teams may lack necessary skills; They may interact in ways that hinder adherence	Organise teams in which roles are defined and they have a shared goal	Interviews or focus group discussion with targeted healthcare professionals
• **Referral processes**	Processes for transferring patients and communication between different levels of care, between health and social services, and between the targeted healthcare professionals and targeted patients	What referral processes and communication is needed between different levels of care, between health and social services, and between the targeted healthcare professionals and targeted patients? What changes are needed to adhere?	Poor communication; Inappropriate referrals	Structured referral sheets; involvement of consultants in primary care educational activities; Tailored patient information; Patient-held medical records	Interviews or focus group discussion with targeted healthcare professionals and with patients; Observation
**5. INCENTIVES AND RESOURCES**					
• **Availability of necessary resources**	The extent to which the resources that are needed to adhere are available	What resources are needed to adhere, including: financial and human resources, facilities, equipment and supplies, and technical capacity? Are they available?	Necessary resources may not be available	Provide the necessary resources; Reduce the need for additional resources or their cost; Task shifting	Routinely collected data regarding resources; Interviews or focus group discussion with targeted healthcare professionals and managers
• **Financial incentives and disincentives**	The extent to which patients, individual health professionals and organisations have financial incentives or disincentives to adhere	What financial incentives and disincentives do the patients, individual health professionals and organisations have to adhere?	There may be financial disincentives that hinder adherence	Remove or modify the financial disincentives; Provide financial incentives	Interviews or focus group discussion with targeted healthcare professionals and with patients

Determinants	Definitions	Questions	Examples of specific factors[1]	Examples of related implementation strategies	Methods for identifying the determinants
• **Nonfinancial incentives and disincentives**	The extent to which patients, individual health professionals and organisations have nonfinancial incentives or disincentives to adhere	What nonfinancial incentives and disincentives do the patients, individual health professionals and organisations have to adhere?	Disincentives or a lack of incentives for health professionals - e.g. personal recognition or appreciation (from managers, colleagues or the community), continuing education (opportunities to participate in educational activities), working conditions (facilities, equipment or security), career development (possibilities to specialise or be promoted), management (working relationship with managers) – can hinder adherence	Remove or modify disincentives; Provide incentives	Interviews or focus group discussion with targeted healthcare professionals and with patients
• **Information system**	The extent to which the information system facilitates or hinders adherence	How does the information system facilitate or hinder adherence?	Limitations of the information system may restrict the ability of the targeted healthcare professionals to adhere, e.g. it might not be possible to identify patients in need of follow-up or recall	Improve the information system; Provide appropriate incentives to record needed information	Interviews or focus group discussion with targeted healthcare professionals and managers
• **Quality assurance and patient safety systems**	The extent to which existing quality assurance or patient safety systems facilitate or hinder adherence	How do the existing quality assurance or patient safety systems (or the lack of these) facilitate or hinder adherence?	Limitations or lack of quality assurance or patient safety systems may hinder adherence	Establish or improve a quality assurance or patient safety system; Adapt implementation strategies to work within the constraints of the existing systems	Interviews or focus group discussion with targeted healthcare professionals and managers
• **Continuing education system**	The extent to which the continuing education system facilitates or hinders adherence	How does the continuing education system facilitate or hinder adherence?	The continuing education system may hinder adherence	Modify the continuing education system; Adapt implementation strategies to work within the constraints of the existing system	Interviews or focus group discussion with targeted healthcare professionals and managers
• **Assistance for clinicians**	The extent to which clinicians have the assistance they need to adhere	Do clinicians have the assistance they need to adhere, such as checklists, patient information, decision aids, decision support or clinical supervision?	Clinicians may not have assistance needed to help them adhere	Provide needed assistance, such as checklists, patient information, decision aids, decision support or clinical supervision	Interviews or focus group discussion with targeted healthcare professionals

Determinants	Definitions	Questions	Examples of specific factors[1]	Examples of related implementation strategies	Methods for identifying the determinants
**6. CAPACITY FOR ORGANISATIONAL CHANGE**					
• **Mandate, authority, accountability**	The mandate, authority and accountability for making necessary changes	What organisational changes are needed and who has the mandate and authority to make necessary changes? Who is accountable, to whom and how?	It may not be clear who has the mandate or authority to make changes; There may be a lack of accountability	Allocate or reallocate authority to make relevant decisions; Monitor organisational changes; Formally appoint implementation leaders; Use external change agents; Formal agreements regarding accountability; Increase transparency	Interviews or focus group discussion with targeted healthcare professionals and managers
• **Capable leadership**	The extent to which clinical leaders or managers are capable of making necessary changes	What changes require leadership or management? Are there leaders or managers with the necessary capacity (including knowledge, project management, other necessary skills and time)? Are they engaged and is their leadership or management style suitable?	Leaders or managers may lack awareness or familiarity with the recommendation; They may not have the necessary capacity; They may not be engaged; They may not have a suitable style	Engage leaders or managers in designing and implementing the implementation strategy; Provide external support or training for managers and leaders; Shift or allocate leadership or management responsibilities to someone with a suitable style	Interviews or focus group discussion with targeted healthcare professionals and managers
• **Relative strength of supporters and opponents**	The extent of support and opposition to necessary changes	Who supports and who opposes necessary changes?	Opposition to changes may hinder necessary changes	Engage and persuade opponents; Engage and capitalise on supporters; Facilitate dialogue, understanding of conflicting perspectives and achievement of a consensus	Interviews or focus group discussion with targeted healthcare professionals
• **Regulations, rules, policies**	The extent to which organisational regulations, rules or policies facilitate or hinder necessary changes	How do internal and external organisational regulations, rules or policies facilitate or hinder necessary changes?	Organisational regulations, rules or policies may hinder necessary changes	Change the regulations, rules or policies; Make adaptations to enable adherence to the recommendation within the existing regulations, rules and policies	Interviews or focus group discussion with targeted healthcare professionals and managers
• **Priority of necessary change**	The relative priority given to making necessary changes	How are the necessary changes prioritised relative to other priorities?	The necessary changes may be a low priority	Persuade those responsible to change their priorities; Accept the current priorities and work within those constraints	Interviews or focus group discussion with targeted healthcare professionals and managers

Determinants	Definitions	Questions	Examples of specific factors[1]	Examples of related implementation strategies	Methods for identifying the determinants
• **Monitoring and feedback**	The extent to which monitoring and feedback are needed at organisational level and available to sustain necessary changes (including evaluations of improvement programs)	Are monitoring and feedback needed to sustain necessary changes? If so are they available?	Monitoring and feedback may be needed and not available	Use external support to provide monitoring and feedback; Establish required monitoring and feedback	Interviews or focus group discussion with targeted healthcare professionals and managers
• **Assistance for organisational changes**	The extent to which external support is needed and available for necessary changes	Is external support needed to achieve necessary changes? If so is it available?	External support may be needed and not available	Provide the needed external support	Interviews or focus group discussion with targeted healthcare professionals and managers
**7. SOCIAL, POLITICAL AND LEGAL FACTORS**					
• **Economic constraints on the health care budget**	Limits on the total healthcare budget or its growth	Do economic constraints on the healthcare budget facilitate or hinder changes?	Economic constraints may hinder necessary changes if they entailed a substantial increase to the healthcare budget	Pragmatic consideration of what is feasible; Gradual change; Shifting resources from elsewhere in the healthcare budget; increasing the healthcare budget	Interviews with managers, policymakers and stakeholders
• **Contracts**	The extent to which contracts may affect implementation of necessary changes	Do contracts facilitate or hinder implementation of necessary changes?	Contracts with service providers or enforcement of contracts may not be adequate to ensure implementation of necessary changes	Improvements in contracts, including provision for enforcement	Interviews with managers, policymakers and stakeholders
• **Legislation**	The extent to which legislation may affect implementation of necessary changes	Does legislation (or regulations) facilitate or hinder implementation of necessary changes?	General legislation (e.g. regulating government contracts or working conditions) or health care legislation (e.g. licensing health professionals) may be hinder necessary changes	Modify the option so that it is not in conflict with legislation or modify conflicting legislation	Interviews with managers, policymakers and stakeholders
• **Payer or funder policies**	The extent to which payer or funder policies may affect implementation of necessary changes	Do payer or funder policies facilitate or hinder implementation of necessary changes?	Payer or funder policies may hinder necessary changes	Negotiate with payers or funders to allow or support changes	Interviews with managers, policymakers and stakeholders
• **Malpractice liability**	The extent to which malpractice liability may affect implementation of necessary changes	Do real or perceived risks of malpractice complaints facilitate or hinder implementation of necessary changes?	Perceived or real risks of malpractice complaints may hinder necessary changes or adherence	Provide accurate information to correct misperceptions; Provide support to reduce the risk of inappropriate malpractice complaints	Interviews with managers, policymakers and stakeholders

Determinants	Definitions	Questions	Examples of specific factors[1]	Examples of related implementation strategies	Methods for identifying the determinants
• **Influential people**	The extent to which influential people may affect implementation of necessary changes	Do influential people (outside of the targeted healthcare organisations) facilitate or hinder implementation of necessary changes?	Influential people may be opposed to necessary changes	Engage and persuade opponents; Engage and capitalise on supporters; Facilitate dialogue, understanding of conflicting perspectives and achievement of a consensus	Interviews with managers, policymakers and stakeholders
• **Corruption**	The extent to which corruption may affect implementation of necessary changes	Does corruption facilitate or hinder implementation of necessary changes?	Corrupt behaviour may hinder necessary changes and adherence	Increase transparency and accountability; decrease incentives or factors that motivate corruption	Interviews with managers, policymakers and stakeholders
• **Political stability**	The extent to which political stability may affect implementation of necessary changes	Does political stability or instability facilitate or hinder implementation of necessary changes?	Political changes may hinder necessary changes	Engage stable politicians, civil servants or leaders	Interviews with managers, policymakers and stakeholders

i. Guyatt GH, Oxman AD, Kunz R, Vist GE, Falck-Ytter Y, Schunemann HJ, and the GRADE Working Group. What is 'quality of evidence' and why is it important to clinicians? BMJ 2008; 336:995-8.

ii. Guyatt GH, Oxman AD, Kunz R, Falck-Ytter Y, Vist GE, Liberati A, Schunemann HJ, and the GRADE Working Group. Going from evidence to recommendations. BMJ 2008; 336:1049-51.

iii. Shiffman RN, Dixon J, Brandt C, Essaihi A, Hsiao A, Michel G, O'Connell R. The GuideLine Implementability Appraisal (GLIA): development of an instrument to identify obstacles to guideline implementation. BMC Medical Informatics and Decision Making 2005; 5:23.

iv. Oxman AD, Glasziou P, Williams JW. What should clinicians do when faced with conflicting recommendations? BMJ 2008; 337:a2530.

v. Groll RPTM, Bosch MC, Hulscher MEJL, Eccles MP, Wensing M. Planning and Studying Improvement in Patient Care: The Use of Theoretical Perspectives. Milbank Quarterly 2007; 85:93-138.
