# Supplementary material for: A checklist for identifying determinants of practice: A systematic review and synthesis of frameworks and taxonomies of factors that prevent or enable improvements in healthcare professional practice
Source: Implement Sci. 2013 Mar 23;8:35. doi: 10.1186/1748-5908-8-35 (PMC3617095; doi:10.1186/1748-5908-8-35)
Supplement: Additional file 5 — TICD Worksheet 1: Prioritisation of recommendations. [file 1748-5908-8-35-S5.pdf]

Additional file 5 TICD Worksheet 1: Prioritisation of recommendations

Because clinical practice guidelines, practice parameters, practice policies and other packages of clinical recommendations commonly include many recommendations, it is necessary to focus implementation efforts on those recommendations where there is the greatest potential for benefit.

We suggest that at least two people independently assess and then discuss the importance of focusing implementation efforts on each recommendation by applying the three criteria in the worksheet (consequences of non-adherence, evidence of non-adherence or inequitable adherence, feasibility of recommended practice). Other factors that affect judgements about whether implementing a recommendation is a priority can be noted in the last column together with the overall judgement. No fixed weights are assigned to the three criteria. However, if a recommendation scores low on any of the three criteria, its priority is likely to be low.

We suggest selecting a small number of recommendations as the focus of implementation efforts and no more than five to eight. Because the determinants of practice and potential strategies to address those need to be considered for each recommendation, it becomes increasingly difficult to identify important determinants and to design and deliver an implementation intervention as the number of recommendations increases.

Date: **Your name(s)**:

Guideline:

Targeted health professionals:

Recommendation	Are the consequences of non-adherence serious?*	Is there a large amount of non-adherence or inequitable adherence?*	Is the recommended practice feasible in the targeted settings?*	Is implementing the recommendation a priority?*
1.				
2.				
3.				
4.				
5.				
6.				
7.				
8.				
9.				
10.				
11.				
12.				
13.				
14.				
15.				
16.				

* Use the following scoring system. Comments can be included in each cell following the score; e.g. justifying the judgement or clarifying the reason for uncertainty.

1 = No
2 = Probably not
3 = Uncertain
4 = Probably
5 = Yes
