# Supplementary material for: A checklist for identifying determinants of practice: A systematic review and synthesis of frameworks and taxonomies of factors that prevent or enable improvements in healthcare professional practice
Source: Implement Sci. 2013 Mar 23;8:35. doi: 10.1186/1748-5908-8-35 (PMC3617095; doi:10.1186/1748-5908-8-35)
Supplement: Additional file 6 — TICD Worksheet 2: Initial assessment of determinants. [file 1748-5908-8-35-S6.pdf]

Additional file 6 TICD Worksheet 2 for initial assessment of determinants

This worksheet should be used to:

- *Tailor the TICD checklist to the specific recommendations by dropping irrelevant factors, adding potentially important factors that may be missing, and modifying questions to make them specific for the recommendations*
- *Plan further investigations that are needed to identify and prioritise factors that should be addressed by implementation strategies; including reasons for or against including factors for further investigation, such as uncertainty about their importance or the potential to do something to address them*

We suggest that at least two people independently assess the need for further investigation of each factor for each recommendation being implemented and then discuss their judgements, as with the first worksheet.

Each factor should be considered in relationship to each recommendation that is considered to be a priority for implementation. Reasons for further investigation (e.g. to confirm assumptions for which there is little or no evidence, to clarify characteristics of a factor, to obtain a deeper understanding of a factor, or to gain insights into potential strategies to address a factor) or for not investigating a factor further (e.g. if there already is sufficient evidence or further investigations would be unlikely to lead to any changes in implementation strategies) should be recorded in the corresponding cell for factors where there may be disagreement or a need for clarification. It is not necessary to fill in cells where there is unlikely to be disagreement or a need for clarification. Modifications of the questions in the TICD checklist and plans for further investigation should also be recorded for factors where changes and further investigation are needed.

We suggest maintaining some open ended questions together with questions focused on specific factors that warrant further investigation. For example, one might want to include a question for each domain, such as "Are there any other characteristics of the guidelines that might hinder or facilitate implementing the recommendation?"

Date:

Your name(s):

Recommendations:

1.
2.
3.
4.
5.
6.
7.
8.

Determinants	Investigate further (Check relevant recommendation #'s)[1]	Reasons for further investigation (or for not investigating further)	Proposed modifications of the questions to tailor them to the specific recommendations	Plans for further investigation (e.g. interviews or focus group discussions with people in the target audience or patients)	Comments (including potential strategies to address barriers or capitalise on facilitators)
**1. GUIDELINE FACTORS**					
• Recommendation					
• Quality of evidence supporting the recommendation	1 2 3 4 5 6 7 8 ☐☐☐☐☐☐☐☐				
• Strength of recommendation	1 2 3 4 5 6 7 8 ☐☐☐☐☐☐☐☐				
• Clarity	1 2 3 4 5 6 7 8 ☐☐☐☐☐☐☐☐				
• Cultural appropriateness	1 2 3 4 5 6 7 8 ☐☐☐☐☐☐☐☐				
• Accessibility of the recommendation	1 2 3 4 5 6 7 8 ☐☐☐☐☐☐☐☐				
• Source of the recommendation	1 2 3 4 5 6 7 8 ☐☐☐☐☐☐☐☐				
• Consistency with other guidelines	1 2 3 4 5 6 7 8 ☐☐☐☐☐☐☐☐				
Recommended clinical intervention					

[1] Assign numbers to each of the recommendations being implemented. For each factor, check the number of any recommendation for which further investigation is warranted.

Determinants	Investigate further (Check relevant recommendation #'s)[1]	Reasons for further investigation (or for not investigating further)	Proposed modifications of the questions to tailor them to the specific recommendations	Plans for further investigation (e.g. interviews or focus group discussions with people in the target audience or patients)	Comments (including potential strategies to address barriers or capitalise on facilitators)
• Feasibility	1 2 3 4 5 6 7 8 ☐☐☐☐☐☐☐☐				
• Accessibility of the intervention	1 2 3 4 5 6 7 8 ☐☐☐☐☐☐☐☐				
Recommended behaviour					
• Compatibility	1 2 3 4 5 6 7 8 ☐☐☐☐☐☐☐☐				
• Effort	1 2 3 4 5 6 7 8 ☐☐☐☐☐☐☐☐				
• Troalability	1 2 3 4 5 6 7 8 ☐☐☐☐☐☐☐☐				
• Observability	1 2 3 4 5 6 7 8 ☐☐☐☐☐☐☐☐				
• Other	1 2 3 4 5 6 7 8 ☐☐☐☐☐☐☐☐				
2. INDIVIDUAL HEALTH PROFESSIONAL FACTORS					
Knowledge and skills					
• Domain knowledge	1 2 3 4 5 6 7 8 ☐☐☐☐☐☐☐☐				
• Awareness and familiarity with the recommendation	1 2 3 4 5 6 7 8 ☐☐☐☐☐☐☐☐				
• Knowledge about own practice	1 2 3 4 5 6 7 8 ☐☐☐☐☐☐☐☐				
• Skills needed to adhere	1 2 3 4 5 6 7 8 ☐☐☐☐☐☐☐☐				
Cognitions (including attitudes)					
• Agreement with the recommendation	1 2 3 4 5 6 7 8 ☐☐☐☐☐☐☐☐				
• Attitudes towards guidelines in general	1 2 3 4 5 6 7 8 ☐☐☐☐☐☐☐☐				
• Expected outcome	1 2 3 4 5 6 7 8 ☐☐☐☐☐☐☐☐				
• Intention and motivation	1 2 3 4 5 6 7 8 ☐☐☐☐☐☐☐☐				
• Self-efficacy	1 2 3 4 5 6 7 8 ☐☐☐☐☐☐☐☐				

Determinants	Investigate further (Check relevant recommendation #'s)[1]	Reasons for further investigation (or for not investigating further)	Proposed modifications of the questions to tailor them to the specific recommendations	Plans for further investigation (e.g. interviews or focus group discussions with people in the target audience or patients)	Comments (including potential strategies to address barriers or capitalise on facilitators)
• Learning style	1 2 3 4 5 6 7 8 ☐☐☐☐☐☐☐☐				
• Emotions	1 2 3 4 5 6 7 8 ☐☐☐☐☐☐☐☐				
Professional behaviour					
• Nature of the behaviour	1 2 3 4 5 6 7 8 ☐☐☐☐☐☐☐☐				
• Capacity to plan change	1 2 3 4 5 6 7 8 ☐☐☐☐☐☐☐☐				
• Self-monitoring or feedback	1 2 3 4 5 6 7 8 ☐☐☐☐☐☐☐☐				
• Other	1 2 3 4 5 6 7 8 ☐☐☐☐☐☐☐☐				
3. PATIENT FACTORS					
• Patient needs	1 2 3 4 5 6 7 8 ☐☐☐☐☐☐☐☐				
• Patient beliefs and knowledge	1 2 3 4 5 6 7 8 ☐☐☐☐☐☐☐☐				
• Patient preferences	1 2 3 4 5 6 7 8 ☐☐☐☐☐☐☐☐				
• Patient motivation	1 2 3 4 5 6 7 8 ☐☐☐☐☐☐☐☐				
• Patient behaviour	1 2 3 4 5 6 7 8 ☐☐☐☐☐☐☐☐				
• Other	1 2 3 4 5 6 7 8 ☐☐☐☐☐☐☐☐				
4. PROFESSIONAL INTERACTIONS					
• Communication and influence	1 2 3 4 5 6 7 8 ☐☐☐☐☐☐☐☐				
• Team processes	1 2 3 4 5 6 7 8 ☐☐☐☐☐☐☐☐				
• Referral processes	1 2 3 4 5 6 7 8 ☐☐☐☐☐☐☐☐				
• Other	1 2 3 4 5 6 7 8 ☐☐☐☐☐☐☐☐				
5. INCENTIVES AND RESOURCES					
• Availability of necessary resources	1 2 3 4 5 6 7 8 ☐☐☐☐☐☐☐☐				

Determinants	Investigate further (Check relevant recommendation #'s)[1]	Reasons for further investigation (or for not investigating further)	Proposed modifications of the questions to tailor them to the specific recommendations	Plans for further investigation (e.g. interviews or focus group discussions with people in the target audience or patients)	Comments (including potential strategies to address barriers or capitalise on facilitators)
• Financial incentives and disincentives	1 2 3 4 5 6 7 8<br>☐☐☐☐☐☐☐☐				
• Nonfinancial incentives and disincentives	1 2 3 4 5 6 7 8<br>☐☐☐☐☐☐☐☐				
• Information system	1 2 3 4 5 6 7 8<br>☐☐☐☐☐☐☐☐				
• Quality assurance and patient safety systems	1 2 3 4 5 6 7 8<br>☐☐☐☐☐☐☐☐				
• Continuing education system	1 2 3 4 5 6 7 8<br>☐☐☐☐☐☐☐☐				
• Assistance for clinicians	1 2 3 4 5 6 7 8<br>☐☐☐☐☐☐☐☐				
• Other	1 2 3 4 5 6 7 8<br>☐☐☐☐☐☐☐☐				
**6. CAPACITY FOR ORGANISATIONAL CHANGE**					
• Mandate, authority, accountability	1 2 3 4 5 6 7 8<br>☐☐☐☐☐☐☐☐				
• Capable leadership	1 2 3 4 5 6 7 8<br>☐☐☐☐☐☐☐☐				
• Relative strength of supporters and opponents	1 2 3 4 5 6 7 8<br>☐☐☐☐☐☐☐☐				
• Regulations, rules, policies	1 2 3 4 5 6 7 8<br>☐☐☐☐☐☐☐☐				
• Priority of necessary change	1 2 3 4 5 6 7 8<br>☐☐☐☐☐☐☐☐				
• Monitoring and feedback	1 2 3 4 5 6 7 8<br>☐☐☐☐☐☐☐☐				
• Assistance for organisational changes	1 2 3 4 5 6 7 8<br>☐☐☐☐☐☐☐☐				
• Other	1 2 3 4 5 6 7 8<br>☐☐☐☐☐☐☐☐				
**7. SOCIAL, POLITICAL AND LEGAL FACTORS**					
• Economic constraints on the health care budget	1 2 3 4 5 6 7 8<br>☐☐☐☐☐☐☐☐				
• Contracts	1 2 3 4 5 6 7 8<br>☐☐☐☐☐☐☐☐				

Determinants	Investigate further (Check relevant recommendation #'s)[1]	Reasons for further investigation (or for not investigating further)	Proposed modifications of the questions to tailor them to the specific recommendations	Plans for further investigation (e.g. interviews or focus group discussions with people in the target audience or patients)	Comments (including potential strategies to address barriers or capitalise on facilitators)
• Legislation	1 2 3 4 5 6 7 8 ☐☐☐☐☐☐☐☐				
• Payer or funder policies	1 2 3 4 5 6 7 8 ☐☐☐☐☐☐☐☐				
• Malpractice liability	1 2 3 4 5 6 7 8 ☐☐☐☐☐☐☐☐				
• Influential people	1 2 3 4 5 6 7 8 ☐☐☐☐☐☐☐☐				
• Corruption	1 2 3 4 5 6 7 8 ☐☐☐☐☐☐☐☐				
• Political stability	1 2 3 4 5 6 7 8 ☐☐☐☐☐☐☐☐				
• Other	1 2 3 4 5 6 7 8 ☐☐☐☐☐☐☐☐				
