# Supplementary material for: A checklist for identifying determinants of practice: A systematic review and synthesis of frameworks and taxonomies of factors that prevent or enable improvements in healthcare professional practice
Source: Implement Sci. 2013 Mar 23;8:35. doi: 10.1186/1748-5908-8-35 (PMC3617095; doi:10.1186/1748-5908-8-35)
Supplement: Additional file 7 — TICD Worksheet 3: Prioritisation of determinants. [file 1748-5908-8-35-S7.pdf]

Additional file 7 TICD Worksheet 3: Prioritisation of determinants

This worksheet is intended to be used after investigations of potential factors have been completed. It provides space to note the recommendations to which each factor applies, the likely impact of the factor on adherence and the evidence of the presence of the factor and its likely impact on adherence. We suggest that at least two people assess and discuss the likely impact of each factor, potential implementation strategies and the potential to target implementation strategies (e.g. at specific clinicians or practices). The completed table provides a basis for determining which factors the implementation strategy should be tailored to address.

Date:

Your name(s):

Guideline:

Determinant	Recommendation(s) to which the determinant applies	Likely impact of the determinant	Evidence[1] (of the presence of the determinant and its likely impact on adherence)[2]	Impact score[3]	Potential implementation strategies	Potential to target implementation strategies (e.g. at specific clinicians or practices)

[1] From brainstorming, literature reviews, surveys, interviews, focus groups, routinely collected data, observation)

[2] Consider the potential for people's perceptions of the determinants to be wrong.

[3] Scoring of the likely impact:

-3 = major reduction in adherence
- 2 = moderate reduction in adherence
- 1 = minor reduction in adherence
+ 1 = minor increase in adherence
+2 = moderate increase in adherence
+3 = major increase in adherence
