# Supplementary material for: A checklist for identifying determinants of practice: A systematic review and synthesis of frameworks and taxonomies of factors that prevent or enable improvements in healthcare professional practice
Source: Implement Sci. 2013 Mar 23;8:35. doi: 10.1186/1748-5908-8-35 (PMC3617095; doi:10.1186/1748-5908-8-35)
Supplement: Additional file 8 — TICD Worksheet 4: Development of an implementation strategy. [file 1748-5908-8-35-S8.pdf]

# Additional file 8 TICD Worksheet 4: Development of an implementation strategy

*Prioritised factors, their impact scores and potential implementation strategies should be taken from worksheet 3. There may be more than one implementation strategy for each factor and the same implementation strategy may address more than one factor. In addition, implementation strategies may interact and reinforce each other. We suggest that at least two people then assess the likely impact of each implementation strategy, its feasibility and whether it should be included as part of a package of implementation strategies. The likely impact and feasibility should first be noted (in text) and then scored. Plans for further actions should be recorded in the last column, including any information needs and plans for further development of the strategy.*

**Date**: **Your name(s)**:

**Guideline**:

| Prioritised determinants | Impact score for determinant[1] | Potential implementation strategies[2] | Likely impact of the implementation strategy | Potential impact score for the strategy[3] | Feasibility of the implementation strategy | Feasibility score[4] | Should the strategy be targeted (yes/no) | Actions |
|---|---|---|---|---|---|---|---|---|
| | | | | | | | | |
| | | | | | | | | |
| | | | | | | | | |
| | | | | | | | | |
| | | | | | | | | |

[1] *Scoring of the likely impact:
-3 = major reduction in adherence
- 2 = moderate reduction in adherence
- 1 = minor reduction in adherence
+ 1 = minor increase in adherence
+2 = moderate increase in adherence
+3 = major increase in adherence

[2] There may be more than one potential implementation strategy per barrier.

[3] Scoring of the likely impact on improving adherence:
1 = minor increase in adherence
2 = moderate increase in adherence
3 = major increase in adherence

[4] Feasibility score:
1 = low
2 = moderate
3 = high
