# Supplementary material for: A checklist for identifying determinants of practice: A systematic review and synthesis of frameworks and taxonomies of factors that prevent or enable improvements in healthcare professional practice
Source: Implement Sci. 2013 Mar 23;8:35. doi: 10.1186/1748-5908-8-35 (PMC3617095; doi:10.1186/1748-5908-8-35)
Supplement: Additional file 9 — TICD Worksheet 5: Reporting of determinants. [file 1748-5908-8-35-S9.pdf]

Additional file 9 TICD Worksheet 5: reporting of determinants

In worksheet 5 we suggest a structure for reporting determinants of practice in research reports that includes which factors were considered (those in the checklist and any additional ones), which ones were investigated and a reference to a report of the investigation (or a report of the methods that were used), how the factors were prioritised, and a table summarising the main factors, their likely impacts and the evidence supporting that judgement.

Suggested structure for reporting determinants of practice in research reports:

1. The checklist that was used to identify potential determinants + a reference to the checklist
2. The determinants that were investigated + a reference to a report of the investigation
3. How the determinants were prioritised and a table summarising the prioritised determinants

Table

**Determinant (barrier or facilitator)**	**Recommendation(s) for which the determinant is relevant**	**Likely impact of the determinant**	**Evidence (of the presence of the determinant and its likely impact on adherence)**
